# Species-specified VOC emissions derived from a gridded study in the Pearl River Delta, China

**DOI:** 10.1038/s41598-018-21296-y

**Published:** 2018-02-14

**Authors:** Ziwei Mo, Min Shao, Ying Liu, Yang Xiang, Ming Wang, Sihua Lu, Jiamin Ou, Junyu Zheng, Meng Li, Qiang Zhang, Xuemei Wang, Liuju Zhong

**Affiliations:** 1 State Joint Key Laboratory of Environmental Simulation and Pollution Control, College of Environmental Sciences and Engineering, Peking University, Beijing, 100871 China; 2 Department of Mechanical Engineering, The University of Hong Kong, Pokfulam Road, Hong Kong, China; 3 Institute for Environmental and Climate Research, Jinan University, Guangzhou, 511443 China; 4 Beijing Innovation Center for Engineering Science and Advanced Technology (BIC-ESAT), Peking University, Beijing, 100871 China; 5 Jiangsu Key Laboratory of Atmospheric Environment Monitoring and Pollution Control, Nanjing University of Information Science & Technology, Nanjing, 210044 China; 6 School of International Development, University of East Anglia, Norwich, NR4 7TJ United Kingdom; 7 School of Energy and Environment, South China University of Technology, Guangzhou, 510006 China; 8 Ministry of Education Key Laboratory for Earth System Modeling, Center for Earth System Science, Tsinghua University, Beijing, 100871 China; 9 School of Atmospheric Sciences, Sun Yat-Sen University, Guangzhou, 510275 China

## Abstract

This study provides a top-down approach to establish an emission inventory of volatile organic compounds (VOC) based on ambient measurements, by combining the box model and positive matrix factorization (PMF) model. Species-specified VOC emissions, source contributions, and spatial distributions are determined based on regional-scale gridded measurements between September 2008 to December 2009 in the Pearl River Delta (PRD), China. The most prevalent anthropogenic species in the PRD was toluene estimated by the box model to be annual emissions of 167.8 ± 100.5 Gg, followed by m,p-xylene (68.0 ± 45.0 Gg), i-pentane (49.2 ± 40.0 Gg), ethene (47.6 ± 27.6 Gg), n-butane (47.5 ± 40.7 Gg), and benzene (46.8 ± 29.0 Gg). Alkanes such as propane, i-butane, and n-pentane were 2–8 times higher in box model than emission inventories (EI). Species with fewer emissions were highly variable between EI and box model results. Hotspots of VOC emissions were identified in southwestern PRD and port areas, which were not reflected by bottom-up EI. This suggests more research is needed for VOC emissions in the EI, especially for fuel evaporation, industrial operations and marine vessels. The species-specified top-down method can help improve the quality of these emission inventories.

## Introduction

Volatile organic compounds (VOCs) have raised growing public concerns due to their crucial role in the formation of ground-level ozone. Reducing these VOC emissions is essential in the Pearl River Delta (PRD), China, where the region is battling ground-level ozone (O_3_) pollution during the past decade^1^. A 10% increase of annual ozone concentrations was observed, from 48 μg/m^3^ in 2006 to 53 μg/m^3^ in 2015^2^. Quantitative determination of the VOC emissions and their source contributions is needed to develop effective pollution control strategies.

Emission inventory (EI), often compiled using a bottom-up calculation estimate, is a widely-used and easily-defined approach to track VOC emissions at a global scale^3,4^, regional scale^5,6^ and city-scale^7^. A high quality emission inventory has detailed source categories and spatial-temporal variation, requiring considerable amounts of time and money to compile. Even so, developing accurate EI is challenging and fraught with large uncertainties, primarily resulting from non-local emission factors and coarse activity data^8,9^. Unlike other pollutants (e.g., carbon monoxide [CO], sulfur dioxide [SO_2_], nitrogen oxides [NO_x_]), VOC emission estimates are more complex and difficult to accurately detail due to their many varied species and wide array of sources. The individual species emissions might have an order of magnitude difference when allocating non-local source profiles^6,10^.

Validations of emission inventories have been conducted using different top-down approaches, such as applying inverse modeling techniques to satellite retrieval^11,12^ and ground-based observations^13^, as well as applying an emission ratio (ER) method to ground-level online measurements^14,15^ and gridded measurements^16,17^. Satellite retrieval of glyoxal showed EI might underestimate aromatic emissions by a factor of 10–20^12^, while a recent study claimed there was a good agreement between the satellite retrievals and EI in the PRD^18^.

The PRD is a rapidly urbanized and highly industrialized region in China, recognized as a hotspot of VOC emissions^19,20^. Much effort has been put to advance our understandings of VOC emissions in this region^5,21,22^. A highly resolved (with a grid size of 3 km × 3 km) speciated VOC emission inventory in the PRD is established^5,23,24^, and several national-scale inventories also cover this region^6,10,25^. The total anthropogenic VOC emissions in the PRD were estimated to be 850.7 Gg in 2006 by Zheng *et al*.^5^, 1174.0 Gg in 2010 by Yin *et al*.^23^, 814.8 Gg in 2006 by Intercontinental Chemical Transport Experiment Phase B (INTEX-B) project^19^, 1156.6 Gg in 2008 and 1283.8 Gg in 2010 by Multi-resolution Emission Inventory (MEIC)^6^. However, large difference in emission estimates exists for key VOC species in most current PRD EI. For example, benzene emissions varied from 8 Gg to 54 Gg and toluene from 44 Gg to 181 Gg reported by Regional Emission Inventory in Asia (REAS)^25^, Representative Concentration Pathways Scenario 2.6 (RCP2.6)^26^, Ou *et al*.^24^, and MEIC^6^. An inverse modelling technique was deployed to constrain the emissions of benzene (44 Gg) and toluene (131 Gg) in this region, indicating that INTEX-B largely underestimated (by a factor of ten) the toluene emissions in the PRD for 2006. These results show that emissions of individual species still exhibit large discrepancies among EI estimates, while the uncertainties for total VOC emissions may appear to be reduced. Source contributions to total emissions were also evaluated by chemical mass balance (CMB) and positive matrix factorization (PMF) models in the PRD^21,22^. Good agreement was observed for vehicular emissions between EI (with a contribution of 41.5%)^5^ and receptor modelling results (31.2–52.6%)^21,22^. However, large difference was found in contributions of liquefied petroleum gas (LPG) emissions (4.9–16.3% in receptor modelling and 0.4% in EI)^5,21^. A comprehensive validation of species-specified VOC emissions is needed to gain a better understanding of the priority species and key sources in the PRD.

The main purpose of this study is to evaluate species-specified VOC emissions using ground-based measurement data from gridded sampling campaigns between 2008 and 2009 in the PRD. The speciated VOC emissions are calculated using a simple chemistry box model, and their source apportionments and spatial distributions are determined by PMF model. Combining the results from box model and PMF model, this study provides a new approach to examine species-individual VOC emissions, and validate the bottom-up emission inventory at a level of species emissions and their spatial distribution at a regional scale. The ground observation-derived EI can give suggestions and directions of re-evaluating the long-term emission estimates and updating the bottom-up EI, which provide support for policy-making and benefit air quality management.

## Results and Discussions

### Species-specified VOC emissions and their uncertainty

#### Species emissions

**Figure 1:**
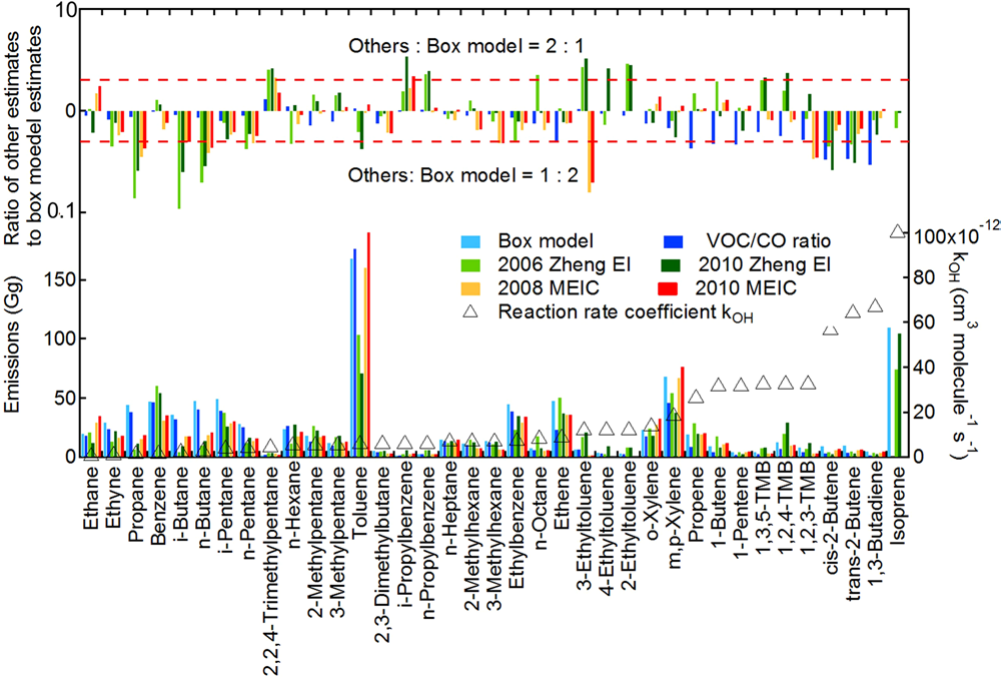
Comparisons of annual VOC emissions (bottom) and their difference (upper) between box model and VOC/CO ratio, bottom-up emission inventories (The species were listed in the sequence of reaction rate coefficient, *k*_OH_).

Figure 1 shows the annual emissions (average emissions for the year of 2008 and 2009) of species-specified VOC in PRD estimated by box-model, VOC/CO ratio method, and available emission factor-derived EI, including 2006 Zheng EI^5^, 2010 Zheng EI^24^, 2008 MEIC and 2010 MEIC^6^. Emission inventories which only reported the total emissions or limited VOC species were not considered^25,26^. Here, we look into the comparisons of inert species and different groups of VOC species. For CO emissions which have less uncertainty in EI and their estimates by box model were not significantly affected by chemical term, the box model result (4.1 × 10^3^ Gg) was proved reasonable and reliable, which was comparable with inventory estimates (3.8 × 10^3^–4.9 × 10^3^ Gg). Halocarbon emissions estimated by box model were in reasonable agreement (difference <60%) with VOC/CO ratio calculation and the previous study^16^ (Table S1 in the Supplementary Information, SI).

For NMHCs, the most prevalent compound found was toluene, whose annual emissions in the PRD were 167.8 ± 100.5 Gg and 176.6 ± 132.6 Gg estimated by box model and VOC/CO ratio method, respectively. The higher emissions estimated by VOC/CO method mainly attributed to a larger regression slope (VOC/CO ratio) because of high concentrations occasionally measured during sampling. These results are significantly larger than those in Zheng EI (103.1 Gg in 2006 and 70.7 Gg in 2010), and much closer to the MEIC (160.2 Gg in 2008 and 193.7 Gg in 2010). The toluene were likely to be underestimated by Zheng EI, which was also indicated by the inverse modelling (131 Gg)^13^. Benzene, m/p-xylene and o-xylene emissions were well within the range of EI estimates (SI Table S1). Their emissions were in good agreement between different methods and also justified by space-based observations^18^. Species with minor emissions such as 3-ethyltoluene, 2-ethyltoluene, and 1,3,5-trimethylbenzene were 3 times higher in Zheng EI, while 3-ethyltoluene was 6 times lower in MEIC. For C_3_-C_5_ alkanes, such as propane, i-butane, n-butane, i-pentane and n-pentane, their annual emissions were slightly higher in the box model (27.9 Gg–49.2 Gg), compared with VOC/CO ratio method (25.1 Gg–40.6 Gg) which has larger uncertainty in species emissions from non-combustion sources; however, the comparable EI estimates are much lower (3.9 Gg–37.5 Gg). These alkanes are major components of fuel evaporation^27^, and should be under priority scrutiny in EI due to difficulties in developing accurate activity and fuel use data of fugitive emissions. The emissions of ethene and propene were estimated to be 47.6 ± 27.6 Gg and 19.2 ± 10.7 Gg by box model, which were comparable with those in EI, but much larger than those (23.2 ± 13.7 Gg and 8.3 ± 4.8 Gg) by VOC/CO ratio method. Moreover, the emissions of trans-2-butene and cis-2-butene calculated by box model were 2–4 times higher than those from VOC/CO ratio method and EI. A main reason that VOC/CO ratio method would underestimate the alkenes with high reaction rate with OH radicals (Fig. 1) because of neglecting their chemical losses.

Comparing the EI for different years, it was shown that there were −56% to 260% differences between 2006 Zheng EI and 2010 Zheng EI, while 0–50% increases for most species from 2008 MEIC to 2010 MEIC (SI Table S1). The total anthropogenic VOC (AVOC) emissions were 1174.0 Gg in 2010 Zheng EI, larger than 850.7 Gg in 2006 Zheng EI. In contrast, sum of the 36 species emissions were lower in 2010 Zheng EI (621.0 Gg; 52.9% of total AVOC emissions) than 2006 Zheng EI (666.3 Gg; 78.3%). Sum of the species emissions accounted for about 55% of the total AVOC emissions for both 2008 MEIC (1156.6 Gg) and 2010 MEIC (1283.8 Gg). The main reasons for these changes were the upgrading of source profiles in Zheng EI, while increased economic activity and implementation of emission control measures in MEIC which used an identical source profile dataset.

Comparing the box model estimates (derived from 2008–2009 measurements) with bottom-up EI for a similar year (2010 Zheng EI and 2008 MEIC), discrepancies still exist. Most noticeable is the higher estimates (up to a factor of eight) of alkane emissions (such as propane, i-butane, n-butane) by box model. This suggests that VOC emissions estimated by the top-down and bottom-up methods could significantly differ from each other, which needs further examination using inter-complementary methods.

#### Uncertainty

The speciated emissions estimated by box model have an uncertainty of 60–100% (SI Figure S1). The contributions from VOC concentration uncertainty and the mixing layer height uncertainty were relatively constant for all species, together accounting for 70–80% of the total uncertainty. These are generally considered the main source of the uncertainty for species emissions estimated by box model. For the high reactive species such as isoprene and 1,2,3-trimethylbenzene, their uncertainty (with a value of approximately 100%) relates to the OH concentration; the uncertainty for less reactive species is more associated with the wind speed. The largest uncertainty is found for C_4_-C_5_ alkanes with a value of over 80–85%. For the species with moderate reactivity, the uncertainty is in the range of 60% to 80%.

### Source contributions to total VOCs and key species

#### Source contribution to total VOC emissions

Table 1 compares the VOC source emissions and their contributions in the PRD derived from this study and emission inventories. Eight sources were identified by PMF with their source profiles and tracer species illustrated in SI Figure S2 and Table S2. The PMF-resolved sources included gasoline vehicle exhaust (25.9%), diesel vehicle exhaust (5.4%), gasoline evaporation (10.9%), industrial emissions (18.2%), solvent usage (23.2%), LPG evaporation (7.9%), stationary fuel combustion (5.7%), and biogenic emissions and background (2.8%), which were comparable to the results reported by Yuan *et al*.^22^. Since source classifications in PMF and EI differ from each other, source contributions in PMF and inventories were combined into similar categories for comparison (SI Tables S3 and S4).

**Table 1:** Comparisons of source emissions (unit: Gg yr^−1^) and their contributions (%) in this study and emission inventories (EI).

Category	This study	2006 Zheng EI	2010 Zheng EI	Category	This study	2008 MEIC	2010 MEIC
Gasoline vehicle exhaust	231.1	344.4	228.9	Transportation	279.1	147.4	106.6
Diesel vehicle exhaust	48.0	28.6	4.3				
Gasoline evaporation	97.8	20.8	16.8	Residential	70.7	34.0	36.7
LPG evaporation	70.7	2.2	—				
Industrial emissions	162.6	6.6	90.6	Industry	467.4	460.1	588.6
Solvent usage	207	240.8	248.8				
Stationary fuel combustion	51.2	22.8	31.6	Power	51.2	1.8	1.8
Sum of species emissions	868.4	666.3	621.0	Sum of species emissions	868.4	643.3	733.7

The emissions of gasoline vehicle exhaust (GVE) were similar between box model results and 2010 Zheng EI, while much lower than those in 2006 Zheng EI. On the other hand, emissions of diesel vehicle exhaust (DVE) estimated in this study were close to 2006 Zheng EI, while much larger than those in 2010 Zheng EI. One reason for the smaller contribution of DVE in 2010 Zheng EI is because the on-road DVE was categorized into the GVE in 2010 Zheng EI (SI Table S3). The box model results of gasoline and LPG evaporation were 4–6 times of those in Zheng EI, suggesting that fugitive emissions was probably a problematic source in EI. Industrial emissions varied significantly between this study and Zheng EI. As PRD is known as the “world’s manufacturing capital”, industrial emissions were recommended to re-evaluate in Zheng EI, which might miss the activity data from the small factories in the PRD. Solvent usage and stationary fuel combustion emissions agreed reasonably between this study and Zheng EI, implying that their contributions are relatively well quantified. For the MEIC inventories, only four categories (transportation, industry, residential, and power) were available for comparison. The emissions of transportation, residential, and power sectors in 2008 and 2010 MEIC were much less, while industry sector contributed much larger than those in this study. MEIC inventories have larger industry’s contributions in the PRD region, and less contributions from transportation, residential, and power sectors. Thus, the total overall estimates from previous EI are comparable, although the specifics of which sectors contribute how much show some significant differences.

#### Source contribution to key species emissions

**Figure 2:**
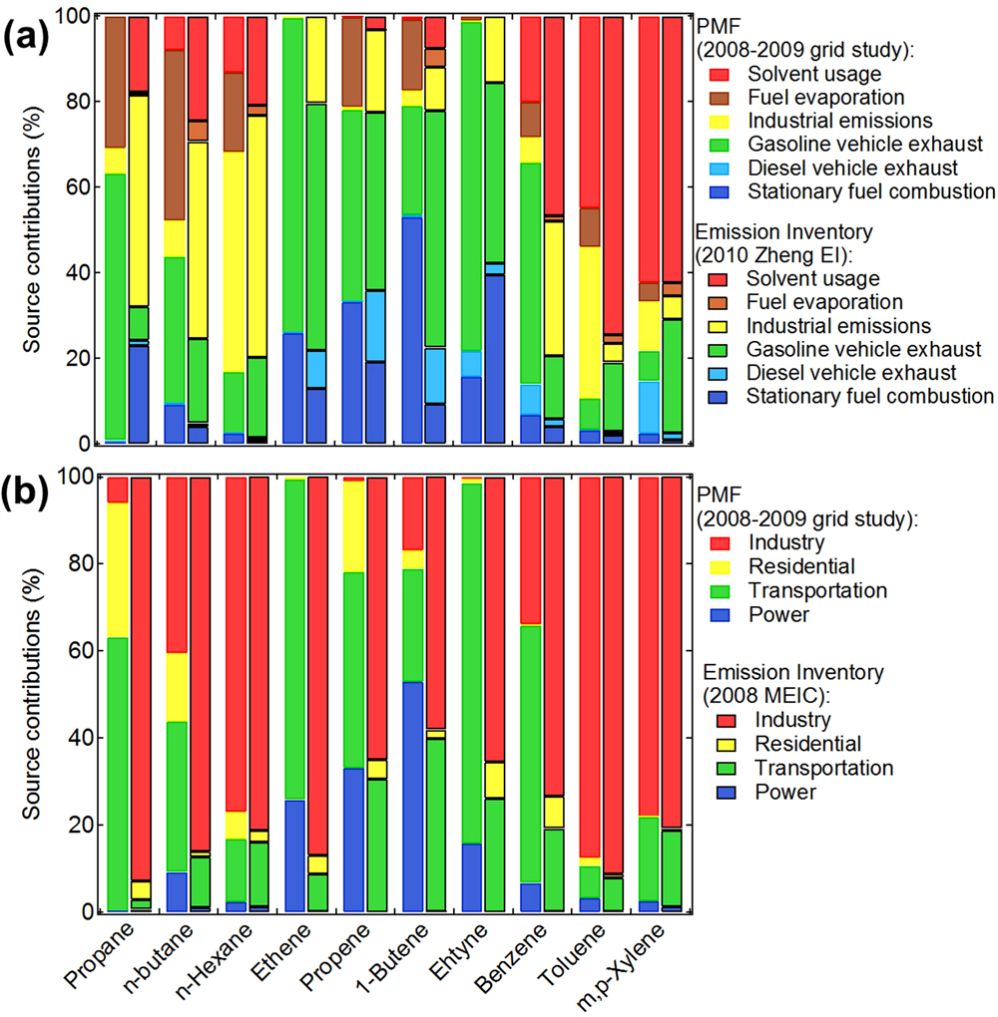
Comparisons of source contributions to key species between the PMF results and 2010 Zheng EI and 2008 MEIC.

Figure 2 compares the source contributions to key VOC species emissions between PMF results and available inventories (2010 Zheng EI and 2008 MEIC were used). The 10 key species had large emissions and represent different VOC categories. Obvious difference was observed for propane and n-butane (Fig. 2a). GVE and fuel evaporation were major sources in PMF while industrial emissions was the dominant in 2010 Zheng EI. Stationary fuel combustion was the major source for 1-butene in the PMF, whereas in Zheng EI it was found predominantly in GVE. Since GVE included fuel combustion in engines, the 1-butene in GVE were probably part of the combustion source in PMF. Regarding aromatics, solvent usage was the most important contributor to toluene and m,p-xylene in Zheng EI, but PMF results showed industrial emissions were also a significant source for toluene in PRD. The GVE dominated benzene emissions in PMF, while solvent usage and industrial emissions contributed most in 2010 Zheng EI. Benzene emissions should be further examined in EI which was strictly controlled in solvent and industrial manufacturing in China during the past few years.

In comparing the PMF results with the 2008 MEIC (Fig. 2b), significant differences were found for propane, ethane, ethyne and benzene. Industry was identified as the largest contributor of these compounds in 2008 MEIC while transportation was the most significant in PMF. Since these compounds are abundant species in the vehicular source profiles^28^, the differences were attributed to lower estimates of transportation contribution in MEIC.

### Spatial variations of VOC emissions

#### Total VOC and key species emissions

**Figure 3:**
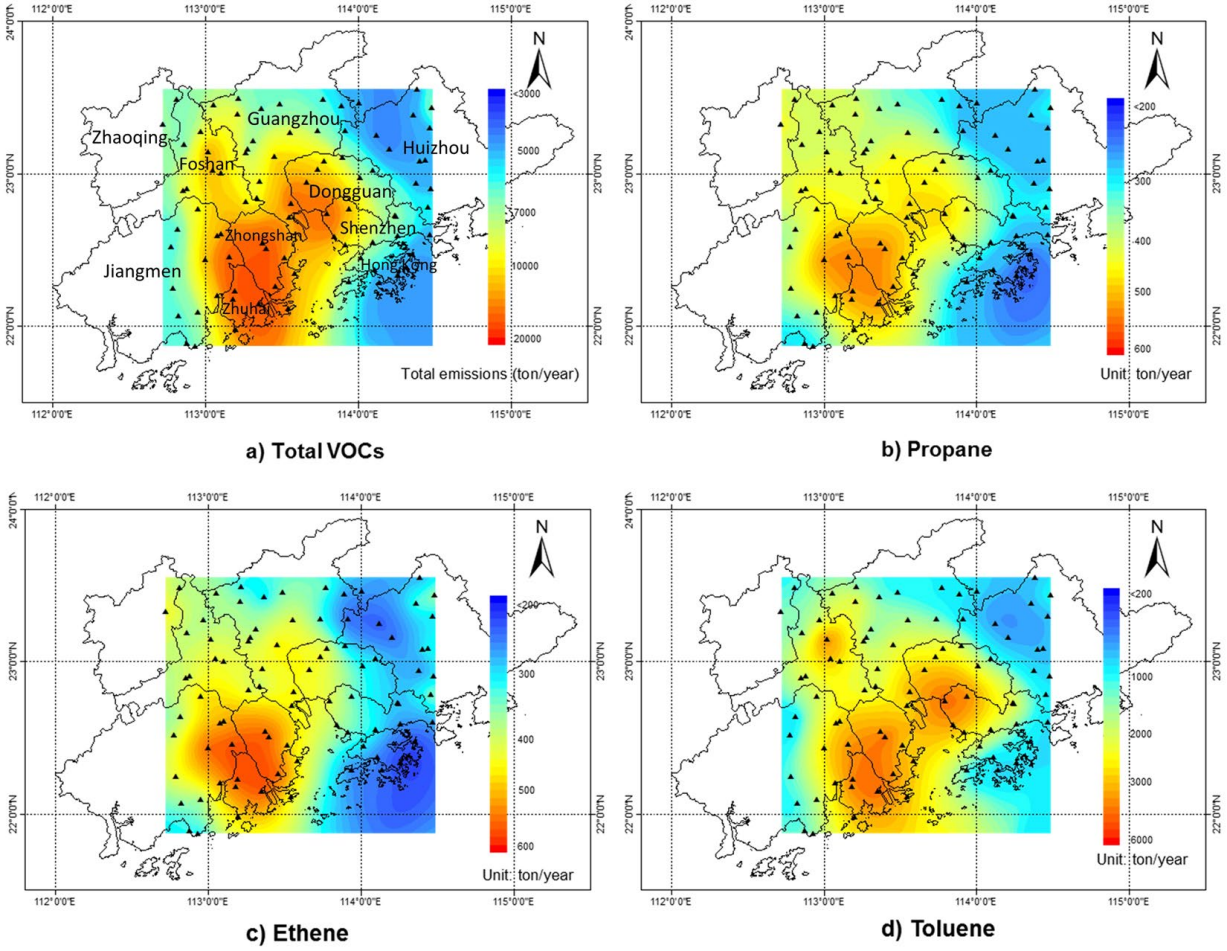
Contour maps of emissions of (**a**) sum of the VOC species, (**b**) propane, (**c**) ethane, and (**d**) toluene estimated by box model. (The maps were generated by ArcGIS Desktop version 10.0, ESRI, Redlands, CA, USA; URL, http://www.esri.com).

The spatial distributions of box model-estimated emissions for total VOCs and three key species (propane, ethene and toluene) are illustrated in Fig. 3. The total emissions estimated by box model were distributed into 84 grids based on the PMF modeling results. Here the spatial distributions of VOC emissions in PRD were assumed to be similar to those of VOC ambient concentrations. This assumption is reasonable for the highly reactive species such as 1,3-butadiene and trans-2-butene because their chemical lifetime (SI Table S5) were less than the transport time in each grid. The spatial characteristics of moderately reactive species were reasonable, given their uncertainty within several grids. For the less reactive species, the chemical lifetime was much longer than their transport time in each grid. This might result in large uncertainty of the spatial distribution.

As shown in the Fig. 3a, two hotspots of total VOC emissions (sum of the species considered in this study) were found: one was at Zhongshan and Zhuhai located in the southwestern PRD; the other was at the junction of three cities (Guangzhou, Dongguan and Shenzhen), located near the Pearl River Estuary (Central PRD). The former was most likely attributable to various emissions from small manufacturing workshops scattered over the region of Zhongshan and the Gaolan Port of Zhuhai. The latter hotspot, around the Pearl River Estuary, is where large ports such as Nansha Port, Huangpu Port and Humen Port are busy working and still being expanded. This port activity can produce a large amount of VOCs from vessels and containers. Also, at Dongguan and Shenzhen, many industrial factories were present including printing, shoemaking, and electronic painting, which significantly contributed to local VOC emissions. A small hotspot was identified at Foshan, which was a highly urbanized and industrialized city with VOC emissions resulting from vehicular and industrial sources. Compared with the spatial patterns of Zheng EI and MEIC (SI Figures S3 and S4), significant emissions from populated and industrialized cities (i.e. Guangzhou-Dongguan-Shenzhen) were captured in both bottom-up and top-down methods, but strong emissions in the southwestern PRD (Zhongshan-Zhuhai) were identified only by box model. This study indicated that some unknown sources of VOC emissions might exist in Zhongshan and Zhuhai, but were not included in current inventories. Marine vessels might also contribute to VOC emissions around the Pearl River Estuary, but they had not been adequately documented in previous inventories^5,23^.

Spatial patterns of propane, ethene, and toluene emissions are shown in Fig. 3(b–d), respectively. Other key species are provided in SI Figure S5. Propane and ethene emission shared a similar spatial distribution, with southwestern PRD as their emission hotspot. In addition to Zhongshan-Zhuhai, Jiangmen was found to contribute significantly to propane and ethene. This is probably because of the LPG used for cooking and their fugitive losses in this area^22^. Three hotspots of toluene emissions were recognized. It is reasonable that Foshan, Dongguan, Shenzhen, and Zhongshan are the most industrialized cities in the PRD, and the higher toluene emissions at Zhuhai might come from the ship-painting and marine vessel emissions.

#### Source emissions

**Figure 4:**
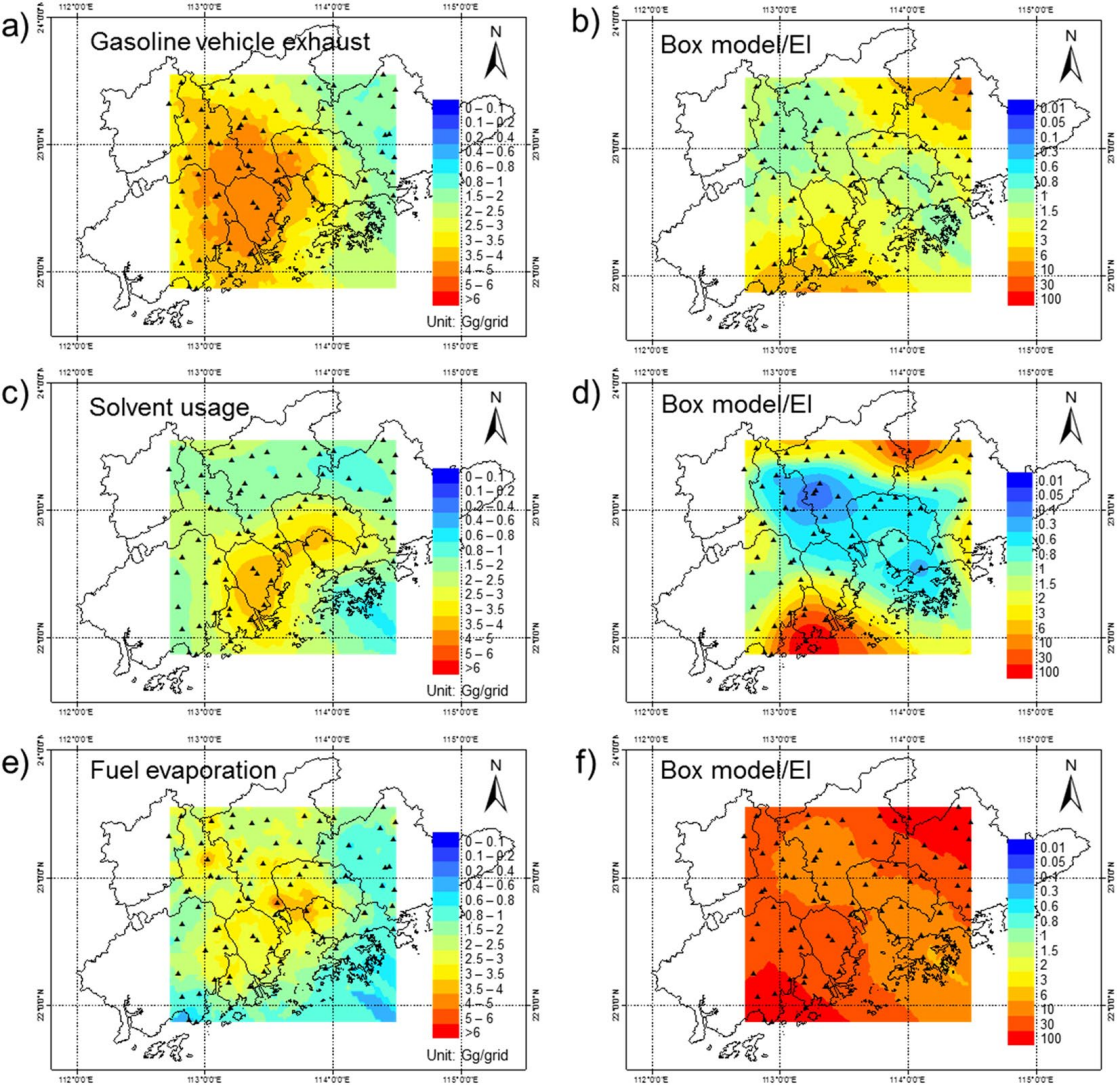
Contour maps of VOC emissions estimated in this study from (**a**) gasoline vehicle exhaust, (**c**) solvent usage, and (**e**) fuel evaporation and their difference (**b**,**d**,**f**) from 2010 Zheng EI. (The maps were generated by ArcGIS Desktop version 10.0, ESRI, Redlands, CA, USA; URL, http://www.esri.com).

The spatial variations of the emissions from gasoline vehicle exhaust, solvent usage and fuel evaporation calculated by box model are shown in Fig. 4a,c and e. Their differences between this study and 2010 Zheng EI are shown in Fig. 4b,d and f. Other sources are provided in SI Figure S6. Emissions from GVE mainly distributed in the center of PRD, connecting the major cities of Guangzhou, Foshan, Zhongshan and Dongguan (Fig. 4a). Not much difference in the spatial variation of GVE emissions was observed between this study and EI, implying that GVE emission distributions were relatively well predicted. For solvent usage, significant emissions were identified in Zhongshan and the nearby areas of Dongguan and Shenzhen. (Fig. 4c). It is reasonable because many industrial factories are located in these areas. Compared with EI, the solvent usage emissions were calculated to be lower in the downtown area of Guangzhou and Shenzhen in this study (Fig. 4d), while they were higher in the northeastern and southwestern parts. Fuel evaporation emissions were higher in Foshan and port areas near the Pearl River Estuary. This probably due to the leakage of vehicular emissions and marine vessel emissions. A whole area of lower emissions from fuel evaporation was estimated in EI compared with the top-down method (Fig. 4f), suggesting the need for revising estimate for fuel evaporation emissions in the PRD.

## Methods

### VOC gridded measurement program

The gridded sampling programs were conducted between 2008 and 2009. A square domain of approximately 200 km × 200 km, covering the major urban areas of the PRD, was divided into 100 square grid cells with sides 20 km in length. Eliminating the grids over water, a total of 84 land sites were selected for VOC sampling. Sampling was conducted 3–42 m above ground, and away from local emission sources to avoid the influence of direct emissions from roadways and industrial sources. Sampling site locations were chosen to represent well-mixed air at each grid area. The site locations, sampling heights and their background information are shown in Figure S7 and Table S6. Whole-air samples were collected simultaneously at all sampling sites using 2-L stainless steel canisters with a duration of 2 minutes^22,29^. Four sampling days with calm weather conditions were selected in September, 2008, March, September, and December in 2009. On each sampling day, two samples were collected at 5 am and 10 am at each site, in order to capture VOC levels both during the nighttime and daytime.

Inter-comparisons of chemical analysis for CO, four halocarbons, and a total of 38 non-methane hydrocarbon (NMHC) species were conducted in laboratories of University of California, Irvine (UCI), Guangzhou Institute of Geochemistry (GIG) and Peking University (PKU)^17,21,30^. At PKU, air samples were concentrated using a three-stage cryofocusing pre-concentration system (Entech 7100, Entech Instruments, USA) and transferred into a gas chromatography–mass spectroscopy/flame ionization detector (GC-MS/FID) system for analysis (GC, HP-7890A; MSD, HP-5975C). This system used a Dean Switch™ to introduce the effluent into a DB-624 column with an MSD to separate and analyze C_4_–C_12_ hydrocarbons and halocarbons and a PLOT (Al/KCl) column with a FID to measure C_2_–C_4_ hydrocarbons. Canisters were connected to another GC-FID for CO analysis. Technical details on the PKU laboratory and the gridded sampling program are described elsewhere^31–34^.

### Emission estimates using top-down approaches

VOC emissions can be estimated using a steady-state zero-dimensional box-model, *Q*/*S* = (*c* − *b*) × *uH*/*L* + *cH*/*τ*, with assumptions that the pollutants are well-mixed inside the box, the mixing height is constant, and only the chemical reactions of VOC species with OH radicals are considered in the daytime^35,36^. A modified box model was adopted in this study, which considered vertical profiles of VOC concentrations. Based on the mass balance of species, the emissions can be calculated as Equation (1):

1 $$\frac{Q}{S}={\int }_{0}^{H}\{\frac{[c(h)-b(h)]\times u}{L}+\frac{c(h)}{\tau }\}dh$$

where *Q* is the emission rate (unit: g s^−1^), *u* is the wind speed (m s^−1^), *L* is the length of the box (m), *h* is the height in vertical dimension (m). *H* is the box height (m), defined as the mixing layer height, *S* is the area of the box bottom (m^2^), *c* (*h*) is the concentration of VOC species at *h* height (μg m^−3^), *b* (*h*) is the background concentration of VOC species at *h* height (μg m^−3^), *τ* is the lifetime of VOC species, determined as 1/(*k*_*OH*_ [*OH*]) (*k*_*OH*_ is reaction rate coefficient for the reaction of VOC species with OH and [*OH*] is the OH concentration).

The vertical structure was divided into 24 layers, which was retrieved from the MM5 model (SI Table S7). Since the reactive species have significant gradient in vertical distribution, the vertical profiles of these species were reconstructed using Equation (2), based on the vertical transport time and chemical lifetime of VOC species.

2 $${\sigma }_{i,voc}={\sigma }_{i,inert}\times (1-\frac{(d{h}_{i}/H)\times {\tau }_{T}}{{\tau }_{C}})$$

where *σ*_*i*, *voc*_ is the dimensionless parameter of VOC concentration at vertical layer *i* normalized by the ground-level concentration, *σ*_*i*, *inert*_ is the dimensionless parameter of inert species normalized by its ground-level concentration. τ_*T*_ is the transport time scale from the ground surface to the boundary layer top, specified as ${\tau }_{T}=\frac{{\sigma }_{z}^{2}}{4{K}_{z}}$ (where ${{\sigma }_{z}}^{2}=\frac{2{K}_{z}x}{\bar{u}}$, *σ*_z_ is the vertical dispersion coefficient, *K*_z_ is the eddy diffusivity)^37^, τ_*C*_ is the chemical lifetime of VOC species, *dh*_*i*_ is the thickness of the vertical layer *i*, *H* is the mixing layer height. The reconstructive vertical profiles of VOC species were compared with the measured vertical profiles^38^, as shown in SI Figure S8. The calculated profiles agreed reasonably with the measured profiles, particularly in the near-ground regions.

The whole domain of the sampling grids was treated as the bottom of the box, with a length (*L*) of 200 km and an area (*S*) of 200 × 200 km^2^. Mixing layer height (*H*) is 441 m–538 m at nighttime, and 513 m–1174 m in the daytime, retrieved from the Global Data Assimilation System (GDAS) of the US National Center for Environmental Prediction (NCEP) (ftp://arlftp.arlhq.noaa.gov/pub/archives/gdas1). The VOC concentrations (*c*) at 5:00 am and 10:00 am were used for nighttime and daytime calculations, respectively. Note that the concentration measured at 5:00 am and 10:00 might biase the results. However, the uncertainty was assumed to be within the uncertainty of average concentration of the four sampling events. The background concentrations (*b*) were assumed as the minimum concentrations at all sampling sites in each campaign. Wind speeds (*u*) were 2–4 m s^−1^ according to the sampling records. OH concentrations were taken as 2–5 × 10^6^ molecule cm^−3^ in daytime, estimated from OH observations in 2006 Program of Regional Integrated Experiments of Air Quality over Pearl River Delta (2006 PRIDE-PRD)^39^. The chemical reactions between VOCs and OH were neglected at nighttime. As only VOC emissions of the whole PRD region are available in emission inventories, speciated VOC emissions calculated by box model, covering an area of 3.4 × 10^4^ km^2^ (eliminating the grids at sea), should be converted to the emissions of the whole PRD (5.5 × 10^4^ km^2^) for comparison. For anthropogenic source-dominated species, their emissions determined from box model were scaled by the gross domestic product (GDP) ratio of the box domain to the whole PRD area, which is 0.82^40^. For biogenic VOCs, represented by isoprene, emission estimates were divided by the forest coverage ratio between above two area, which is 0.57^41^. The summer time is relatively long in PRD and usually overlaps the autumn time. Therefore, VOC emissions for summer and autumn (June–November) were derived from the average value of sampling campaigns in September, 2008 and 2009. The spring time (March–May) and winter time (December–February) emissions were based on sampling campaigns in March, 2009 and December, 2009, respectively.

The annual emissions estimated by box model were determined to be the average emissions for the years of 2008 and 2009.

The total uncertainty from the input parameters in box model was calculated using the error propagation formula in Equation (3):

3 $${s}_{i}=\sqrt{{s}_{c}^{2}+{s}_{H}^{2}+{s}_{u}^{2}+{s}_{[OH]}^{2}}$$

where *s*_*i*_ is the total uncertainty of species *i*, *s*_*c*_, *s*_*H*_, *s*_*u*_, and *s*_*[OH]*_ are the standard deviations of estimated emissions by applying the uncertainties of measured concentration *c*, mixing layer height *H*, wind speed *u*, and OH concentration [*OH*], respectively. The uncertainties of these parameters were determined by the standard deviations of values in the four sampling campaigns. Uncertainties of measured concentrations of different VOC species among the campaigns were determined to be in the range of 20–120%, wind speed within 25%, mixing layer height within 50%, and OH concentration within 50%. We noted that the uncertainties in the box model estimates also arose from other aspects such as small sampling size and sampling representativeness. Quantitatively determination of these uncertainties are difficult and high spatial and temporal resolution of measurements are needed to reduce the uncertainty.

The emissions ratio (ER) method using CO as a reference tracer (referred as “VOC/CO ratio method”) has been widely applied to estimates of VOC emissions in China^15–17^. The basic principle is that if the ratio of VOC enhancement to CO enhancement above background level is determined, VOC emissions are derived by multiplying VOC/CO and CO emissions. Limitations of this method were mentioned in previous studies^15,42^ and details about the emission ratio method can be found in SI. In this work, the VOC/CO method was used as a supplement of VOC estimates for the purpose of comparing the results derived from box models.

### Source apportionment by PMF

The U.S Environmental Protection Agency’s (EPA) PMF 5.0 model was applied to allocate the source contributions to total VOC emissions^43–45^. All the data from eight campaign events were integrated into a sufficiently large dataset to ensure stable and reliable source identification in PMF. A total of 30 species were selected for the PMF applications, all readily detectable in the atmosphere and often viewed as tracers in source identifications (SI Table S8). Detailed introduction of the PMF principle and discussions of applying multiple-site dataset to PMF can be found in SI, Yuan *et al*.^22^ and Dumanoglu *et al*.^46^.

## Electronic supplementary material

Supplementary Information
