# Supplementary material for: Species-specified VOC emissions derived from a gridded study in the Pearl River Delta, China
Source: Sci Rep. 2018 Feb 14;8:2963. doi: 10.1038/s41598-018-21296-y (PMC5813039; doi:10.1038/s41598-018-21296-y)
Supplement: Supplementary file 1 — Supplementary Information [file 41598_2018_21296_MOESM1_ESM.doc]

# Supplementary Information

Species-specified VOC emissions derived from a gridded study in the Pearl River Delta, China

Ziwei Mo^1,2^, Min Shao^3,1*^, Ying Liu^1,4*^, Yang Xiang^1^, Ming Wang^5^, Sihua Lu^1^, Jiamin Ou^6^, Junyu Zheng^3,7^, Meng Li^8^, Qiang Zhang^8^, Xuemei Wang^3,9^, Liuju Zhong^3^

1 State Joint Key Laboratory of Environmental Simulation and Pollution Control, College of Environmental Sciences and Engineering, Peking University, Beijing 100871, China

2 Department of Mechanical Engineering, The University of Hong Kong, Pokfulam Road, Hong Kong, China

3 Institute for Environmental and Climate Research, Jinan University, Guangzhou 511443, China

4 Beijing Innovation Center for Engineering Science and Advanced Technology (BIC-ESAT), Peking University, Beijing 100871, China

5 Jiangsu Key Laboratory of Atmospheric Environment Monitoring and Pollution Control, Nanjing University of Information Science & Technology, Nanjing 210044, China

6 School of International Development, University of East Anglia, Norwich, NR4 7TJ, United Kingdom

7 School of Energy and Environment, South China University of Technology, Guangzhou 510006, China

8 Ministry of Education Key Laboratory for Earth System Modeling, Center for Earth System Science, Tsinghua University, Beijing 100871, China

9 School of Atmospheric Sciences, Sun Yat-Sen University, Guangzhou 510275, China

*** Correspondence to:**

Prof. Min SHAO

Institute for Environmental and Climate Research, Jinan University, Guangzhou 511443, China

Tel: +86-10-62757973; Fax: +86-10-62757973

Email: [mshao@pku.edu.cn](mailto:mshao@pku.edu.cn)

Dr. Ying LIU

College of Environmental Sciences and Engineering, Peking University, Beijing 100871, China

Tel: +86-10-62757973; Fax: +86-10-62757973

Email: yingliu@pku.edu.cn

Table S1 Annual species-specified VOC emissions estimated by box model, VOC/CO ratio, and bottom-up emission inventories (unit: Gg yr^-1^).

|  |  |  |  |  |  |  | Difference (%) between box model and EI | | | |  | |  | |  |
| --- | --- | --- | --- | --- | --- | --- | --- | --- | --- | --- | --- | --- | --- | --- | --- |
| Species | Box-model ^(a)^ | CO ratio ^(a)^ | 2006 Zheng EI^(b)^ | 2010 Zheng EI ^(c)^ | 2008 MEIC^(d)^ | 2010 MEIC^(d)^ | 2006 Zheng EI | 2010 Zheng EI | 2008 MEIC | 2010 MEIC | | Difference of Zheng 2006 and 2010 | | Difference of MEIC 2008 and 2010 | |
| CO (×10^3^) | 4.1 ± 2.5 ^(b)^ | 4.4 ± 2.2 | 3.8 | 4.6 | 4.9 | 4.2 | 8 | -11 | -16 | -2 | | 21 | | -14 | |
| Chloroform (CHCl_3_) | 2.1 ± 1.5 | 2.0 ± 1.3 | 0.9 ^(c)^ | 1.1 ^(d)^ | -- | -- | -- | -- | -- | -- | | -- | | -- | |
| Dichloromethane (CH_2_Cl_2_) | 13.6 ± 8.1 | 20.9 ± 15.8 | 8.1 ^(c)^ | 9.8 ^(d)^ | -- | -- | -- | -- | -- | -- | | -- | | -- | |
| Trichloroethylene (C_2_HCl_3_) | 8.8 ± 9.9 | 8.0 ± 7.1 | 7.7 ^(c)^ | 9.3 ^(d)^ | -- | -- | -- | -- | -- | -- | | -- | | -- | |
| Tetrachloroethylene (C_2_Cl_4_) | 4.8 ± 4.5 | 5.4 ± 4.2 | 2.6 ^(c)^ | 3.2 ^(d)^ | -- | -- | -- | -- | -- | -- | | -- | | -- | |
| Ethane | 19.9 ± 12.3 | 17.8 ± 9.6 | 20.6 | 12.1 | 29.4 | 34.7 | -3 | 64 | -32 | -43 | | -41 | | 18 | |
| Propane | 44.0 ± 28 | 38.2 ± 23.4 | 6.1 | 11.4 | 15.5 | 18.8 | 621 | 286 | 184 | 134 | | 87 | | 21 | |
| i-Butane | 35.7 ± 29.4 | 32.2 ± 20.5 | 3.9 | 8.9 | 17.3 | 17.7 | 815 | 301 | 106 | 102 | | 128 | | 2 | |
| n-Butane | 47.6 ± 40.7 | 40.6 ± 30.3 | 9.4 | 13.5 | 18.3 | 20.6 | 406 | 253 | 160 | 131 | | 44 | | 13 | |
| i-Pentane | 49.2 ± 40.0 | 39.2 ± 26.1 | 37.5 | 26 | 28.5 | 30.2 | 31 | 89 | 73 | 63 | | -31 | | 6 | |
| n-Pentane | 27.9 ± 23.6 | 25.1 ± 18.8 | 11.7 | 16.4 | 13.4 | 15.9 | 138 | 70 | 108 | 75 | | 40 | | 19 | |
| n-Hexane | 23.8 ± 15.9 | 26.1 ± 24.4 | 11.2 | 27.2 | 17.6 | 21.6 | 113 | -13 | 35 | 10 | | 143 | | 23 | |
| n-Heptane | 14.6 ± 9.7 | 13.5 ± 10.7 | 12.1 | 13.7 | 11.8 | 14.6 | 21 | 7 | 24 | 0 | | 13 | | 24 | |
| n-Octane | 7.6 ± 5.7 | 5.7 ± 3.5 | 17.2 | 7.2 | 4.9 | 5.8 | -56 | 6 | 55 | 31 | | -58 | | 18 | |
| 2,3-Dimethylbutane | 5.2 ± 3.2 | 4.0 ± 3.2 | 4.6 | 4.9 | 3.2 | 3.2 | 13 | 6 | 63 | 63 | | 7 | | 0 | |
| 2-Methylpentane | 18.2 ± 12.2 | 13.2 ± 9.4 | 26.2 | 22.6 | 16.9 | 18 | -31 | -19 | 8 | 1 | | -14 | | 7 | |
| 3-Methylpentane | 12 ± 7.3 | 9.5 ± 6.3 | 17.1 | 18.1 | 11.8 | 13 | -30 | -34 | 2 | -8 | | 6 | | 10 | |
| 2-Methylhexane | 11.6 ± 7.3 | 10.4 ± 8.4 | 14.8 | 12.3 | 7.5 | 7.6 | -22 | -6 | 55 | 53 | | -17 | | 1 | |
| 3-Methylhexane | 13.5 ± 9.6 | 12.4 ± 11.0 | 10.6 | 12.9 | 6.5 | 6.5 | 27 | 5 | 108 | 108 | | 22 | | 0 | |
| 2,2,4-Trimethylpentane | 1.3 ± 1.0 | 1.7 ± 1.8 | 3.4 | 3.5 | 2.8 | 2 | -62 | -63 | -54 | -35 | | 3 | | -29 | |
| Ethene | 47.6 ± 27.6 | 23.2 ± 13.7 | 50.4 | 36.9 | 35.8 | 36.1 | -6 | 29 | 33 | 32 | | -27 | | 1 | |
| Propene | 19.2 ± 10.7 | 8.3 ± 4.8 | 28.6 | 19.9 | 19.4 | 20.2 | -33 | -4 | -1 | -5 | | -30 | | 4 | |
| 1-Butene | 9.1 ± 6.6 | 4.3 ± 2.9 | 17.6 | 8 | 11 | 11.7 | -48 | 14 | -17 | -22 | | -55 | | 6 | |
| trans-2-Butene | 9.8 ± 7.1 | 3.3 ± 2.1 | 4.6 | 3 | 5.8 | 6.5 | 113 | 227 | 69 | 51 | | -35 | | 12 | |
| cis-2-Butene | 9.3 ± 7.2 | 3.1 ± 1.8 | 4.1 | 2.4 | 5.9 | 6.8 | 127 | 288 | 58 | 37 | | -41 | | 15 | |
| 1,3-Butadiene | 4.4 ± 3.5 | 1.3 ± 1.3 | 3.6 | 2.6 | 3.7 | 4.6 | 22 | 69 | 19 | -4 | | -28 | | 24 | |
| Isoprene | 109.5 ± 109.2 | -- | 74.1 | 104.6^(g)^ | -- | -- |  |  |  |  | |  | |  | |
| 1-Pentene | 3.9 ± 3.4 | 1.8 ± 1.2 | 4.2 | 2.5 | 4.1 | 4.4 | -7 | 56 | -5 | -11 | | -40 | | 7 | |
| Ethyne | 29.0 ± 18.8 | 23.8 ± 13.6 | 12.9 | 22 | 16.6 | 17.9 | 125 | 32 | 75 | 62 | | 71 | | 8 | |
| Benzene | 46.8 ± 29.0 | 46.4 ± 29.8 | 60.4 | 54.2 | 30.6 | 35.4 | -23 | -14 | 53 | 32 | | -10 | | 16 | |
| Toluene | 167.8 ± 100.5 | 176.6 ± 132.6 | 103.1 | 70.7 | 160.2 | 193.7 | 63 | 137 | 5 | -13 | | -31 | | 21 | |
| Ethylbenzene | 44.7 ± 29.4 | 38.4 ± 31.1 | 22.8 | 34.9 | 29 | 34.2 | 96 | 28 | 54 | 31 | | 53 | | 18 | |
| m,p-Xylene | 68.0 ± 45.0 | 45.8 ± 40.4 | 54.4 | 37.4 | 67 | 76.8 | 25 | 82 | 1 | -11 | | -31 | | 15 | |
| o-Xylene | 23.2 ± 14 | 17.3 ± 14.6 | 23.9 | 17.8 | 27.3 | 32.3 | -3 | 30 | -15 | -28 | | -26 | | 18 | |
| i-Propylbenzene | 1.5 ± 1.0 | 1.4 ± 1.3 | 2.3 | 5.5 | 2.5 | 3.2 | -35 | -73 | -40 | -53 | | 139 | | 28 | |
| n-Propylbenzene | 2.4 ± 1.5 | 2.4 ± 2.4 | 5.5 | 5.9 | 2.3 | 2.6 | -56 | -59 | 4 | -8 | | 7 | | 13 | |
| 3-Ethyltoluene | 6.3 ± 4.6 | 6.6 ± 7.4 | 16.9 | 20.7 | 1 | 1.2 | -63 | -70 | 530 | 425 | | 22 | | 20 | |
| 4-Ethyltoluene | 3.4 ± 2.2 | 3.2 ± 3.5 | 2.5 | 9 | -- | -- | 36 | -62 | -- | -- | | 260 | | -- | |
| 2-Ethyltoluene | 2.8 ± 1.7 | 2.5 ± 2.4 | 8.1 | 7.9 | -- | -- | -65 | -65 | -- | -- | | -2 | | -- | |
| 1,3,5- Trimethylbenzene | 3.8 ± 2.7 | 2.4 ± 2.7 | 7.6 | 8.1 | 3.1 | 3.1 | -50 | -53 | 23 | 23 | | 7 | | 0 | |
| 1,2,4- Trimethylbenzene | 12.4 ± 8.8 | 7.0 ± 7.8 | 19.6 | 29 | 9.6 | 10.1 | -37 | -57 | 29 | 23 | | 48 | | 5 | |
| 1,2,3- Trimethylbenzene | 8.1 ± 7.6 | 4.2 ± 4.2 | 6.8 | 11.9 | 2.7 | 2.8 | 19 | -32 | 200 | 189 | | 75 | | 4 | |
| Sum of species emissions^(e)^ | 868.3 ± 514.7 | 712.9 ± 529.0 | 666.3 | 621 | 643.3 | 733.7 | 30 | 40 | 35 | 18 | | -7 | | 14 | |
| Total AVOCs ^(f)^ | -- | -- | 850.7 | 1174 | 1156.6 | 1283.8 | -- | -- | -- | -- | | 38 | | 11 | |

(a) Year: 2008 – 2009.

(b) Mean ± Uncertainty.

(c) Value from Shao et al. (2011) scaled by the ratio of CO emissions in Shao et al. (2011) (3.3×10^3^ Gg) and in 2006-based emission inventory in Zheng et al. (2009) (3.8 ×10^3^ Gg).

(d) Value from Shao et al. (2011) scaled by the ratio of CO emissions in Shao et al. (2011) (3.3×10^3^ Gg) and in 2010-based emission inventory in Ou et al. (2015) (4.6 ×10^3^ Gg).

(e) Sum of individual species emissions except for isoprene.

(f) Total emissions of anthropogenic VOC reported in the emission inventories.

(g) Value from Wang et al. (2014)

(h) Box model

(i) Negative values are marked in red.

Table S2. Source categories resolved by PMF and their VOC markers.

| Source | Markers | Reference |
| --- | --- | --- |
| Solvent usage | toluene, ethylbenzene, xylenes | Yuan et al. (2010); Liu et al. (2008) |
| Gasoline evaporation | i-pentane, n-butane, i-butene | Liu et al. (2008); Watson et al. (2001) |
| Industrial emissions | m,p-xlyene, n-octane, n-heptane, | Dumanoglu et al. (2014) |
| Biogenic emissions and background | Isoprene, ethane | Guenther et al. (1995) |
| Gasoline vehicle exhaust | C2 species, C4-C5 alkanes | Watson et al. (2001); Yuan et al. (2013) |
| Diesel vehicle exhaust | ethene, ethyne, 1,2,4-trimethylbenzene | Gertler et al. (1996); Liu et al. (2008) |
| LPG evaporation | propane, n-butane, i-butane | Blake and Rowland (1995); Liu et al. (2008) |
| Stationary fuel combustion | ethene, ethyne, benzene, toluene | Liu et al. (2008); Watson et al. (2001) |

Table S3. Re-classifications of source categories in PMF results and 2006/2010 Zheng emission inventories (Zheng et al., 2009; Yin et al., 2015).

| Adjusted categories | PMF categories | 2006-based EI categories | 2010-based EI categories |
| --- | --- | --- | --- |
| Gasoline vehicle exhaust | Gasoline vehicle exhaust | Gasoline vehicle | On-road mobile |
|  |  | motorcycle | |
| Diesel vehicle exhaust | Diesel vehicle exhaust | Diesel vehicle | Non-road mobile |
| Fuel evaporation | Gaslone evaporation | Gasoline evaporation | Fossil fuel storage and transportation |
|  | LPG emissions | LPG | -- |
| Industrial emissions | Industrial emissions | Oil refinery | Industrial processes |
| Solvent usage | Solvent use | Printing | Industrial solvent use |
|  |  | Solvent |  |
|  |  | Building paint | Non-industrial solvent use |
| Stationary fuel combustion | Stationary fuel combustion | Coal burning-boiler | Stationary combustion |
|  |  | Coal burning-residential | |
|  |  | Biomass burning | Biomass burning |
| -- | Biogenic emissions and background | Biogenic source | -- |

Table S4. Re-classifications of source categories in PMF results and MEIC emission inventories (Li et al., 2014).

| MEIC categories | PMF categories |
| --- | --- |
| Transportation | Gasoline vehicle exhaust |
|  | Diesel vehicle exhaust |
| Residential | LPG emissions |
| Industry | Industrial emissions |
|  | Solvent usage |
|  | Gasoline evaporation |
| Power | Combustion |
| -- | Biogenic emissions and background |

Table S5 Chemical lifetime due to OH (2 × 10^6^ - 5 × 10^6^ molecule cm^-3^ )

|  | *K_OH_* (298K) | chemical lifetime |  | |  | *K_OH_* (298K) | chemical lifetime |
| --- | --- | --- | --- | --- | --- | --- | --- |
| Species | (× 10^-12^ cm^3^ molecule^-1^ s^-1^) | Hours |  | | Species | (× 10^-12^ cm^3^ molecule^-1^ s^-1^) | Hours |
| CO | 0.14 | 99.2 - 396.8 | | trans-2-Butene | | 5.78 | 0.2 - 0.9 |
| Ethane | 0.254 | 54.7 - 218.7 | | cis-2-Butene | | 5.2 | 0.2 - 1 |
| Propane | 8.52 | 12.4 - 49.6 | | 1,3-Butadiene | | 5.2 | 0.2 - 0.8 |
| i-Butane | 0.78 | 6.6 - 26.2 |  | | Isoprene | 6.76 | 0.1 - 0.6 |
| n-Butane | 1.12 | 5.9 - 23.5 |  | | 1-Pentene | 6.76 | 0.4 - 1.8 |
| i-Pentane | 26.3 | 3.9 - 15.4 |  | | Ethyne | 4.24 | 17.8 - 71.2 |
| n-Pentane | 2.12 | 3.7 - 14.6 |  | | Benzene | 1.22 | 11.4 - 45.5 |
| n-Hexane | 2.36 | 2.7 - 10.7 |  | | Toluene | 5.63 | 2.5 - 9.9 |
| n-Heptane | 31.4 | 2.1 - 8.2 |  | | Ethylbenzene | 7 | 2 - 7.9 |
| n-Octane | 64 | 1.7 - 6.9 |  | | m,p-Xylene | 18.2 | 0.8 - 3.1 |
| 2,3-Dimethylbutane | 56.4 | 2.4 - 9.6 |  | | o-Xylene | 13.6 | 1 - 4.1 |
| 2-Methylpentane | 3.6 | 2.7 - 10.7 |  | | i-Propylbenzene | 5.8 | 2.4 - 9.6 |
| 3-Methylpentane | 3.8 | 2.7 - 10.7 |  | | n-Propylbenzene | 5.8 | 2.4 - 9.6 |
| 2-Methylhexane | 66.6 | 2.1 - 8.2 |  | | 3-Ethyltoluene | 11.9 | 1.2 - 4.7 |
| 3-Methylhexane | 31.4 | 2.1 - 8.2 |  | | 4-Ethyltoluene | 11.9 | 1.2 - 4.7 |
| 2,2,4-Trimethylpentane | 100 | 3.3 - 13.1 |  | | 2-Ethyltoluene | 11.9 | 1.2 - 4.7 |
| Ethene | 5.2 | 1.6 - 6.5 |  | | 1,3,5-TMB | 32.5 | 0.4 - 1.7 |
| Propene | 6.75 | 0.5 - 2.1 |  | | 1,2,4-TMB | 32.5 | 0.4 - 1.7 |
| 1-Butene | 8.11 | 0.4 - 1.8 |  | | 1,2,3-TMB | 32.5 | 0.4 - 1.7 |

Table S6 Sampling location and their background information.

| **Grid Cell** | **Sampling Location** | **Sampling Position**  **(R=Rooftop; G=Ground)** | **Sampling Height above Ground**  **(m)** | **Area Type** |
| --- | --- | --- | --- | --- |
| A1 | Shuijing Reservoir (水逕水庫) | R | ~9 | Rural |
| A2 | Sankeng Reservoir (三坑水庫) | G |  | Rural |
| A3 | Furongzhang Resort (芙蓉嶂度假村) | G |  | Rural |
| A4 | Huadong Hostel (花東招待所) | G |  | Rural – residential |
| A5 | Guangzhou University Huaruan Software College(廣州大學華軟軟件學院) | R | ~6 | Educational |
| A6 | Paitan Hotel (派潭大酒店) | R | ~12 | Residential |
| A7 | Zhengguo Ecology Village (正果生態村) | R | ~3 | Rural |
| A8 | The People’s Government Buildings of Mazha Town (麻榨鎮人民政府) | G |  | Mixed residential / commercial |
| A9 | The People’s Government Buildings of Gongzhuang Town (公莊鎮人民政府) | G |  | Mixed residential / commercial |
| A10 | Town Government Buildings of Yangqiao Town (楊橋鎮鎮政府) | G |  | Mixed residential / commercial |
| B1 | Plaza of the People’s Government of Sihui City (四會市人民政府廣場) | G |  | Urban – mixed residential / commercial |
| B2 | Yuantan (源潭) | G |  | Rural – residential |
| B3 | Huadu Teacher Training School (花都師範學校) | R | 13 | Mixed residential / commercial |
| B4 | Nanhu Tourism Centre (南湖旅遊中心) | G |  | Tourism |
| B5 | Yunhao Hotel (雲豪度假村) | G |  | Residential |
| B6 | Yingyuan (盈園) | G |  | Rural |
| B7 | Lianhe Reservoir (聯合水庫) | G |  | Rural |
| B8 | Xiangang Reservoir (顯崗水庫) | G |  | Rural |
| B9 | The People’s Government Building of Botang Town (柏塘鎮人民政府) | G |  | Mixed residential / commercial |
| B10 | Konka Technology Ltd.(康佳精密科技有限公司前綠地) | G |  | Mixed residential / commercial |
| C1 | SenlinPark (森林公園) | R | 15 | Urban – mixed residential / commercial |
| C2 | Nanhai Software Technology Park (南海軟件科技園) | R | ~12 | Educational |
| C3^2^ | Guangdong Provincial automatic air quality monitoring station (廣東省環境監測站) | R | 43 | Mixed residential / commercial |
| C4 | Luhu Park (麓湖公園)^*#@ | R | 9 | City |
| C5 | Huangpu Lizhi Park(黃埔荔枝公園) | G |  | Park / sport centre |
| C6 | Datangkou Park (大塘口公園) | G |  | Residential |
| C7 | Dahanyuanlin (大漢園林) | G |  | Residential |
| C8 | Yihe Commercial Plaza(義和商業廣場) | G |  | Residential |
| C9 | Xiapu (下埔)^ | R | 25 | Residential |
| C10 | Huizhou Traffic Police (惠州市交警大隊) | G |  | Mixed residential / commercial |
| D1 | Hecheng Park (荷城公園) | G |  | Residential |
| D2 | Liantang (蓮塘) | R | 15 | Industrial |
| D3 | Huijingcheng (惠景城) | R | 14 | Residential |
| D4 | Panyu Secondary School (番禺中學) | R | 11 | Mixed residential / commercial |
| D5 | Songshan Park (松山公園) | G |  | Residential |
| D6 | Haogang Primary School (豪崗小學)^ | R | ~15 | Mixed residential / commercial / industrial |
| D7 | Changping Railway Park (常平鐵路公園) | G |  | Residential |
| D8 | Lianhu Park (蓮湖公園) | G |  | Residential |
| D9 | Jinguowan (金果灣生態農莊) | R | 8 | Residential |
| D10 | Lenovo Technology Park (Lenovo 科技園) | G |  | Mixed residential / commercial |
| E1 | Gaoming Jizhong (高明紀中) | R | 12 | Urban – mixed residential / commercial |
| E2 | Beihu Hostel (北湖賓館)* | R | ~3 | Residential |
| E3 | Foshan Shunde Dangxiao (佛山順德黨校)^ | R | 16 | Tourist and cultural / educational |
| E4 | Lanhe Plaza (欖核廣場) | G |  | Mixed residential / commercial |
| E5 | Nansha Street Offices Plaza南沙街辦廣場 | G |  | Mixed residential / commercial |
| E6 | Shayicun Park (沙一村公園) | G |  | Residential |
| E7 | Cuihu Park (翠湖公園) | G |  | Residential |
| E8 | Longgang Yi Cun (龍崗一村) | G |  | Residential |
| E9 | Longgang Cultural Central Plaza (龍崗文化中心廣場) | G |  | Mixed residential / commercial |
| E10 | Chuanhu Monitoring Station (船湖子站) | R | 10 | Residential |
| F1 | He Cheng Hostel (鶴城旅業) | R | ~12 | Residential |
| F2 | Donghu Park (東湖公園)^ | R | 4.5 | City |
| F3 | Jiangmen Bei Street (江門北街) | R | 15 | Industrial |
| F4 | Elderly Entertainment Centre (老人康乐中心) | R | ~9 | Mixed residential / industrial |
| F5 | Wanqingsha (萬傾沙)^ | R | 12 | Mixed educational / commercial and residential/ industrial |
| F6 | Bihaiwan Golf Club (碧海灣哥爾夫球場) | R | ~3 | Resort |
| F7 | Lixiang (荔香) | R | 14 | Mixed residential / commercial |
| F8 | Lizhi Park (荔枝公園)^ | R | ~6 | City |
| F9 | Yantian (鹽田) | R | 21 | Mixed residential / commercial |
| F10 | Dongxing Hostel (東星賓館) | R | ~12 | Residential |
| G1 | Zhishan Park (址山公園) | G |  | Residential |
| G2 | Zhenxing Park (振興公園) | G |  | Residential |
| G3 | Muzhou Town Centre Primary School (睦洲鎮中心小學) | G |  | Residential |
| G4 | Zimaling Park (紫馬嶺公園)^ | R | 10.2 | Mixed residential/commercial |
| G5 | Zhongshan Memorial Secondary School District Demonstration Meteorological Station (中山紀念中學區域示範氣象站) | G |  | Residential |
| G6 | *非採樣網格(水面區域)* |  |  |  |
| G7 | Yuen Long (元朗) | R | 25 | New town – residential |
| G8 | Tsuen Wan (荃灣)^*#@ | R | 17 | Urban – mixed residential / commercial / industrial |
| G9 | Tap Mun (塔門) | R | 11 | Background – rural |
| G10 | *非採樣網格(水面區域)* |  |  |  |
| H1 | Ningcheng Park (寧城公園) | G |  | Residential |
| H2 | Yamen Jiaobeishi Primary School (崖門交貝石小學) | R | ~9 | Agricultural / residential |
| H3 | Doumen Park (斗門公園)* | G |  | Residential |
| H4 | Tanzhoushan Park (坦洲山公園) | G | ~2 | Mixed residential / commercial |
| H5 | Tangjia (唐家)^ | R | 15 | Mixed educational / commercial and residential / industrial |
| H6 | *非採樣網格(水面區域)* |  |  |  |
| H7 | Tung Chung (東涌) | R | 21 | New town – residential |
| H8 | Hong Kong Island Central/Western (中西區) | R | 18 | Urban – residential / commercial |
| H9 | HKUST (香港科大) | R | 15 | Educational |
| H10 | *非採樣網格(水面區域)* |  |  |  |
| I1 | The People’s Court of Doushan Town (斗山鎮人民法庭) | G |  | Agricultural |
| I2 | Fudou Spa Hotel (富都溫泉度假村) | R | ~13 | Agricultural / resort |
| I3 | Wansheng Villaeg Club(萬盛鄉村俱樂部) | R | ~6 | Agricultural / resort |
| I4 | Baitengshan Park (白滕山公園) | G |  | Rural |
| I5 | Jinfeng Recreational Resort (金灃娛樂山莊) | G | ~3 | Residential |
| I6 | *Water area(水面區域)* |  |  |  |
| I7 | *Water area(水面區域)* |  |  |  |
| I8 | *Water area(水面區域)* |  |  |  |
| I9 | *Water area(水面區域)* |  |  |  |
| I10 | *Water area(水面區域)* |  |  |  |
| J1 | Kejia Resort (客家山莊) | R | ~3 | Rural – residential |
| J2 | Heishawan Bathing Seashore (黑沙灣海濱浴場) | R | ~9 | Rural |
| J3 | Gang Zhong Gang Hotel (港中港酒店) | R | ~20 | Mixed residential / industrial |
| J4 | *Water area(水面區域)* |  |  |  |
| J5 | *Water area(水面區域)* |  |  |  |
| J6 | *Water area(水面區域)* |  |  |  |
| J7 | *Water area(水面區域)* |  |  |  |
| J8 | *Water area(水面區域)* |  |  |  |
| J9 | *Water area(水面區域)* |  |  |  |
| J10 | *Water area(水面區域)* |  |  |  |

Table S7. Vertical Layer Definitions for MM5.

| Level | Sigma | Height(m) | Pressure(mb) | Thickness(m) |
| --- | --- | --- | --- | --- |
| 23 | 0 | 16262.4 | 100 | 2512.1 |
| 22 | 0.052 | 13750.2 | 146 | 2285.1 |
| 21 | 0.12 | 11465.1 | 208 | 1954.6 |
| 20 | 0.2 | 9510.5 | 280 | 1882.6 |
| 19 | 0.3 | 7627.9 | 370 | 1545.5 |
| 18 | 0.4 | 6082.5 | 460 | 1318.8 |
| 17 | 0.5 | 4763.7 | 550 | 957.9 |
| 16 | 0.582 | 3805.8 | 623.8 | 706.8 |
| 15 | 0.648 | 3099 | 683.2 | 542.4 |
| 14 | 0.702 | 2556.6 | 731.8 | 402.1 |
| 13 | 0.744 | 2154.5 | 769.6 | 304.9 |
| 12 | 0.777 | 1849.6 | 799.3 | 278.2 |
| 11 | 0.808 | 1571.4 | 827.2 | 270.7 |
| 10 | 0.839 | 1300.7 | 855.1 | 246.8 |
| 9 | 0.868 | 1053.9 | 881.2 | 208.1 |
| 8 | 0.893 | 845.8 | 903.7 | 187.8 |
| 7 | 0.916 | 658.1 | 924.4 | 176.4 |
| 6 | 0.938 | 481.6 | 944.2 | 142.1 |
| 5 | 0.956 | 339.5 | 960.4 | 109.2 |
| 4 | 0.97 | 230.3 | 973 | 77.3 |
| 3 | 0.98 | 152.9 | 982 | 61.4 |
| 2 | 0.988 | 91.5 | 989.2 | 53.5 |
| 1 | 0.995 | 38 | 995.5 | 38 |
| 0 | 1 | 0 | 1000 | 0 |

Table S8. Comparison of emission ratios (ERs) with Wang et al. (2014), Beijing.

| Species | Wang et al. (2014) | Wang et al. (2014) | Camp_1 | Camp_2 | Camp_3 | Camp_4 | Average |
| --- | --- | --- | --- | --- | --- | --- | --- |
|  | Summer | Winter | Sep. | Mar. | Sep. | Dec. |  |
| Ethane* | 4.34 | 4.41 | 3.80 | 4.44 | 2.69 | 4.12 | 3.76 |
| Ethene* | 4.44 | 5.79 | 2.94 | 5.76 | 5.60 | 6.76 | 5.27 |
| Ethyne* | 3.67 | 3.26 | 3.86 | 5.12 | 6.83 | 7.45 | 5.82 |
| Propane* | 3.9 | 2.1 | 4.98 | 3.02 | 6.76 | 7.34 | 5.52 |
| Propene* | 1.39 | 1.58 | 0.91 | 1.03 | 1.29 | 1.77 | 1.25 |
| i-Butane* | 2.51 | 0.75 | 3.16 | 1.90 | 5.24 | 3.82 | 3.53 |
| n-Butane* | 2.5 | 0.79 | 2.27 | 2.61 | 7.46 | 5.45 | 4.44 |
| 1-Butene* | 0.54 | 0.29 | 0.23 | 0.43 | 0.57 | 0.74 | 0.49 |
| i-Butene | -- | -- | 0.64 | 0.45 | 0.56 | 0.65 | 0.57 |
| trans-2-Butene* | 0.42 | 0.18 | 0.23 | 0.41 | 0.29 | 0.56 | 0.37 |
| cis-2-Butene* | 0.37 | 0.11 | 0.23 | 0.37 | 0.33 | 0.46 | 0.35 |
| i-Pentane* | 1.98 | 0.48 | 2.24 | 2.40 | 5.53 | 3.67 | 3.46 |
| n-Pentane* | 1.2 | 0.36 | 1.11 | 1.19 | 3.44 | 3.11 | 2.21 |
| 1,3-Butadiene | 0.16 | 0.18 | 0.06 | 0.16 | 0.06 | 0.33 | 0.15 |
| 1-Pentene* | 0.1 | 0.05 | 0.27 | 0.11 | 0.11 | 0.18 | 0.17 |
| Isoprene* | 0.05 | 0.06 | 0.45 | 0.15 | 0.17 | 0.26 | 0.26 |
| n-Hexane* | 0.57 | 0.37 | 4.03 | 0.53 | 1.20 | 1.97 | 1.93 |
| n-Heptane* | 0.2 | 0.11 | 0.59 | 0.38 | 0.89 | 1.58 | 0.86 |
| n-Octane* | 0.12 | 0.06 | 0.42 | 0.16 | 0.35 | 0.34 | 0.32 |
| 2,3-Dimethylbutane* | 0.18 | 0.03 | 0.13 | 0.26 | 0.22 | 0.56 | 0.29 |
| 2-Methylpentane* | 0.61 | 0.25 | 0.45 | 0.65 | 1.32 | 1.47 | 0.97 |
| 3-Methylpentane* | 0.51 | 0.17 | 0.48 | 0.41 | 0.87 | 1.05 | 0.70 |
| 2-Methylhexane* | 0.14 | 0.08 | 0.32 | 0.41 | 0.64 | 1.25 | 0.66 |
| 3-Methylhexane* | 0.23 | 0.08 | 0.28 | 0.45 | 0.86 | 1.58 | 0.79 |
| 2,2,4-Trimethylpentane | 0.01 | 0 | 0.15 | 0.03 | 0.02 | 0.19 | 0.10 |
| Benzene* | 1.24 | 1.06 | 2.42 | 2.72 | 4.31 | 5.69 | 3.79 |
| Toluene* | 2.41 | 1.2 | 8.19 | 4.76 | 16.94 | 18.96 | 12.21 |
| Ethylbenzene* | 0.97 | 0.33 | 1.54 | 0.82 | 2.71 | 4.17 | 2.31 |
| m,p-Xylene* | 1.56 | 0.53 | 1.65 | 1.10 | 2.68 | 5.58 | 2.75 |
| o-Xylene* | 0.59 | 0.19 | 0.97 | 0.39 | 0.77 | 2.04 | 1.04 |
| i-Propylbenzene | 0.03 | 0.01 | 0.04 | 0.04 | 0.07 | 0.16 | 0.08 |
| n-Propylbenzene* | 0.06 | 0.02 | 0.10 | 0.05 | 0.08 | 0.29 | 0.13 |
| 3-Ethyltoluene* | 0.23 | 0.05 | 0.22 | 0.11 | 0.19 | 0.87 | 0.35 |
| 4-Ethyltoluene | 0.09 | 0.03 | 0.12 | 0.06 | 0.09 | 0.42 | 0.17 |
| 2-Ethyltoluene | 0.09 | 0.02 | 0.10 | 0.06 | 0.07 | 0.30 | 0.13 |
| 1,3,5-TMB | 0.11 | 0.02 | 0.08 | 0.06 | 0.04 | 0.32 | 0.12 |
| 1,2,4-TMB* | 0.35 | 0.06 | 0.25 | 0.16 | 0.15 | 0.92 | 0.37 |
| 1,2,3-TMB | 0.11 | 0.02 | 0.38 | 0.07 | 0.05 | 0.41 | 0.22 |

* species used in the PMF model.

Figure S1. Total uncertainty of VOC emissions and their uncertainty distribution estimated by box model (The species are listed in the sequence of chemical reaction rate coefficient, *k*_OH_).

Figure S2. Source profiles (bar) and distributions of each species among the factors (plus) resolved from the PMF model.


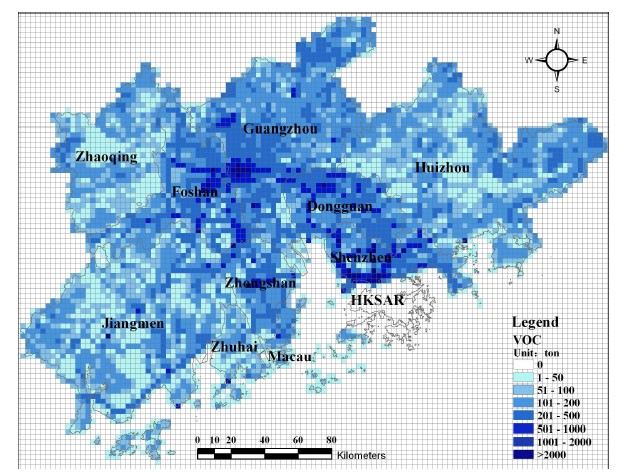

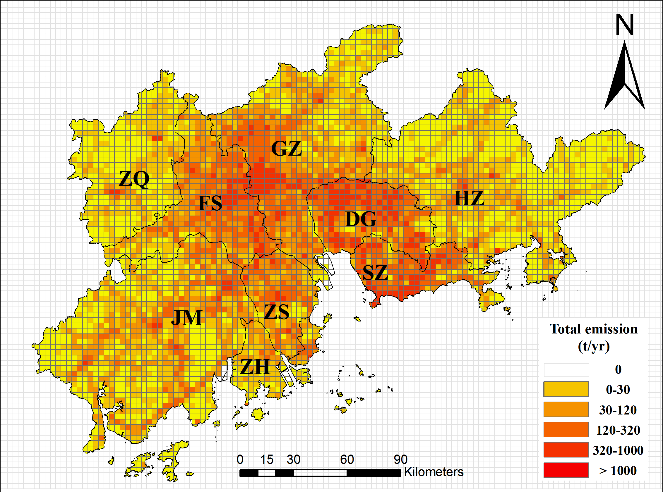


Figure S3. Spatial distributions of 2006-based (left) and 2010-based (right) total VOCs emission inventory estimated by Zheng et al. (2009) and Yin et al. (2015), respectively. (The maps were generated by ArcGIS Desktop version 10.0, ESRI, Redlands, CA, USA; URL, http://www.esri.com)


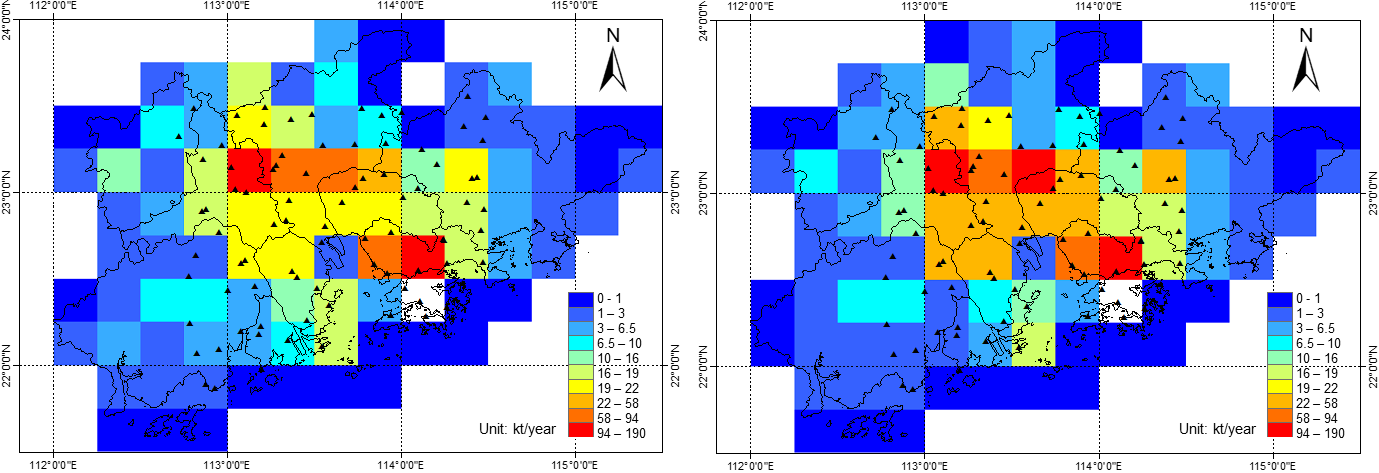


Figure S4. Spatial distributions of 2008-based (left) and 2010-based (right) total VOCs emission inventory retrieved from MEIC. (The maps were generated by ArcGIS Desktop version 10.0, ESRI, Redlands, CA, USA; URL, http://www.esri.com)


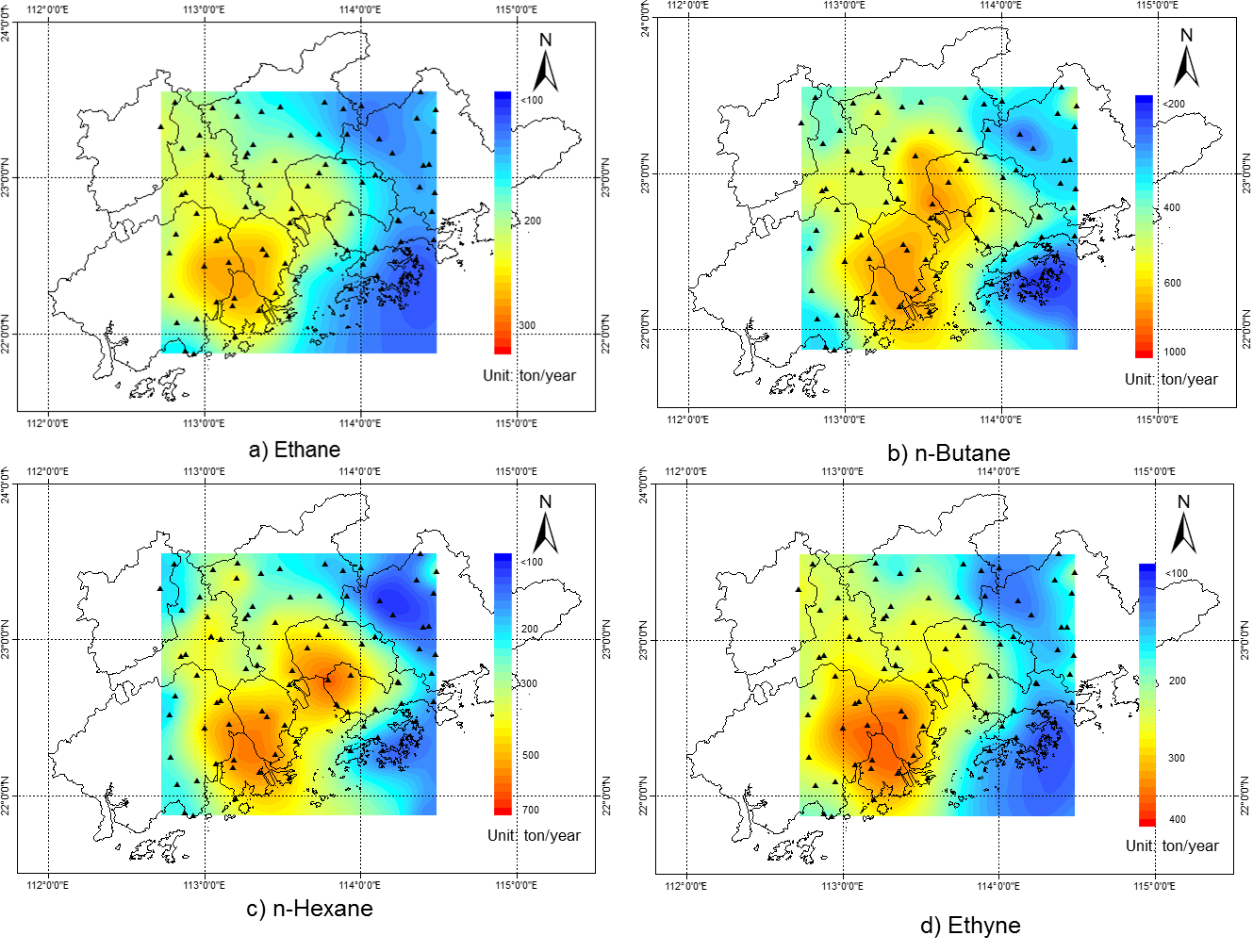


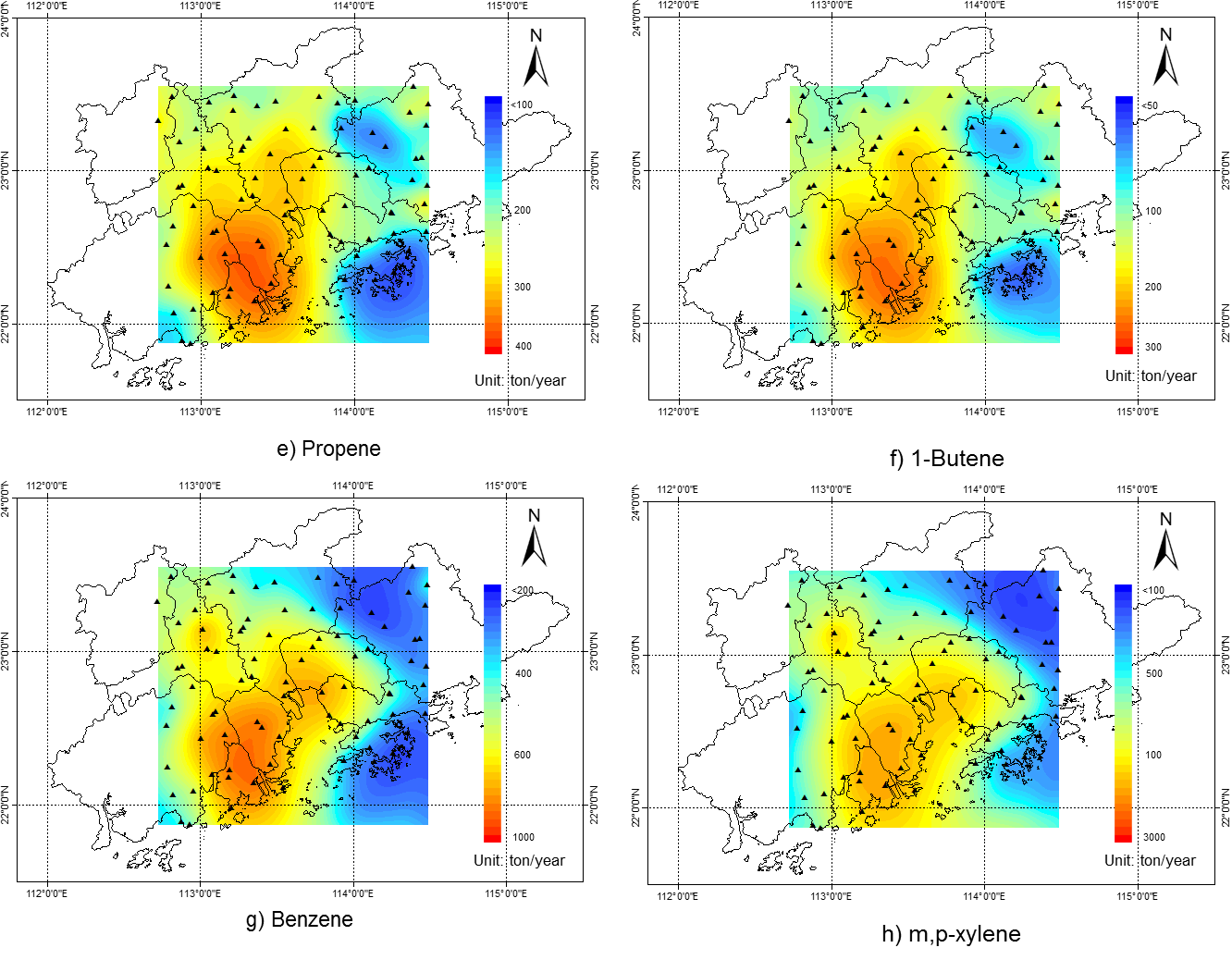


Figure S5. Contour maps of gridded emissions of key species estimated by box model. (The maps were generated by ArcGIS Desktop version 10.0, ESRI, Redlands, CA, USA; URL, http://www.esri.com)


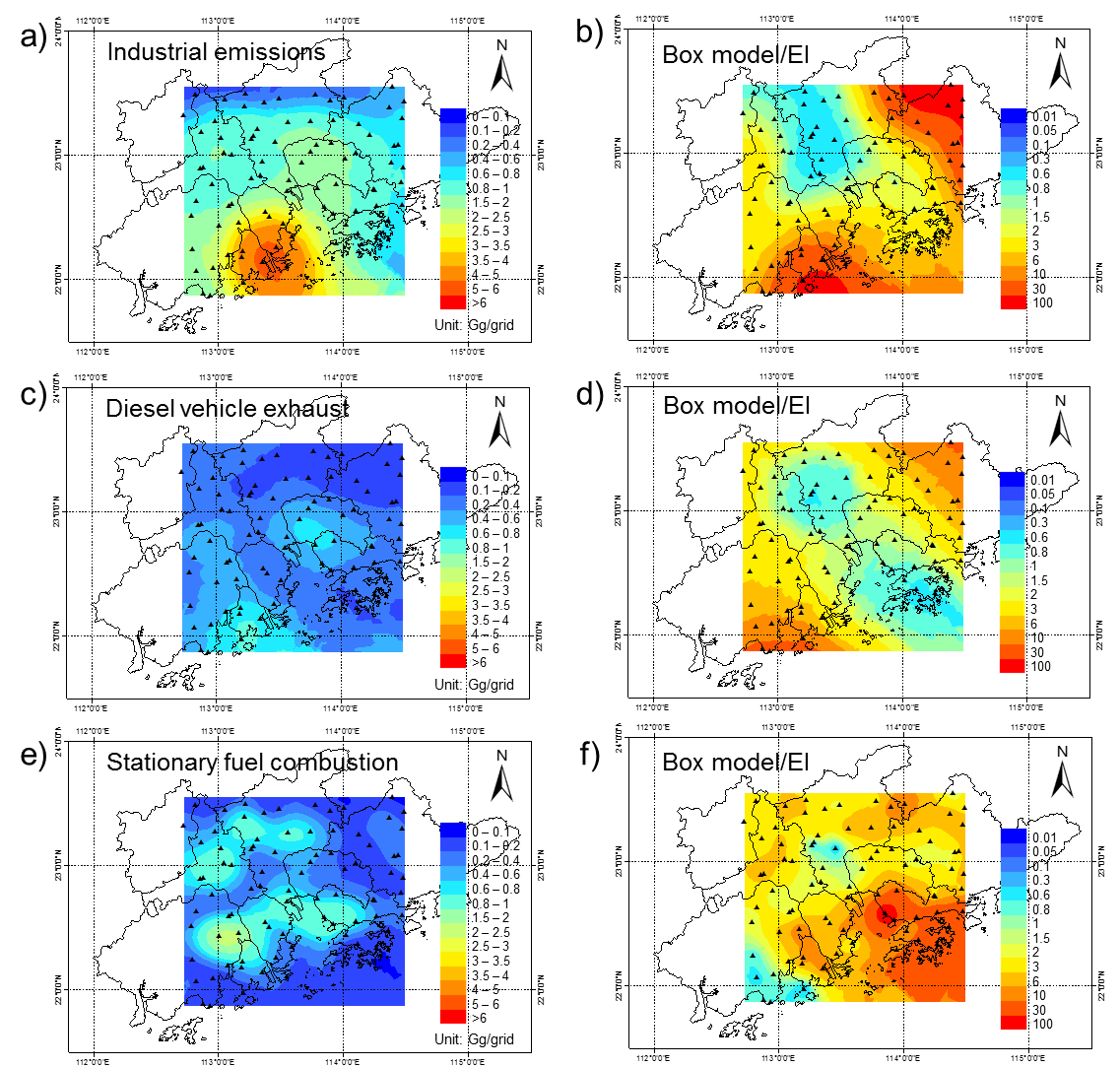


Figure S6. Contour maps of VOC emissions estimated in this study from g) industrial emissions, i) diesel vehicle exhaust, and k) stationary fuel combustion and their difference (b, d, f) from 2010 Zheng EI. (The maps were generated by ArcGIS Desktop version 10.0, ESRI, Redlands, CA, USA; URL, http://www.esri.com)


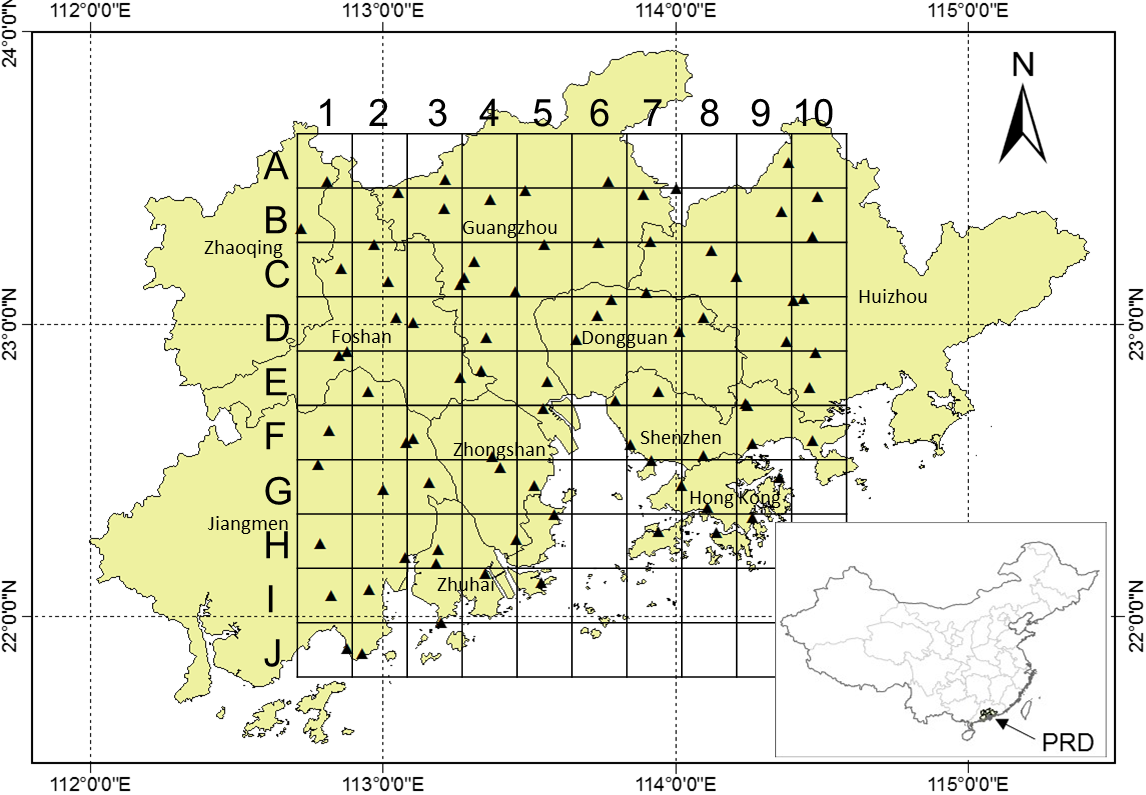


Figure S7. Map of the Pearl River Delta, China and the locations of sampling sites. (The map was generated by ArcGIS Desktop version 10.0, ESRI, Redlands, CA, USA; URL, http://www.esri.com)


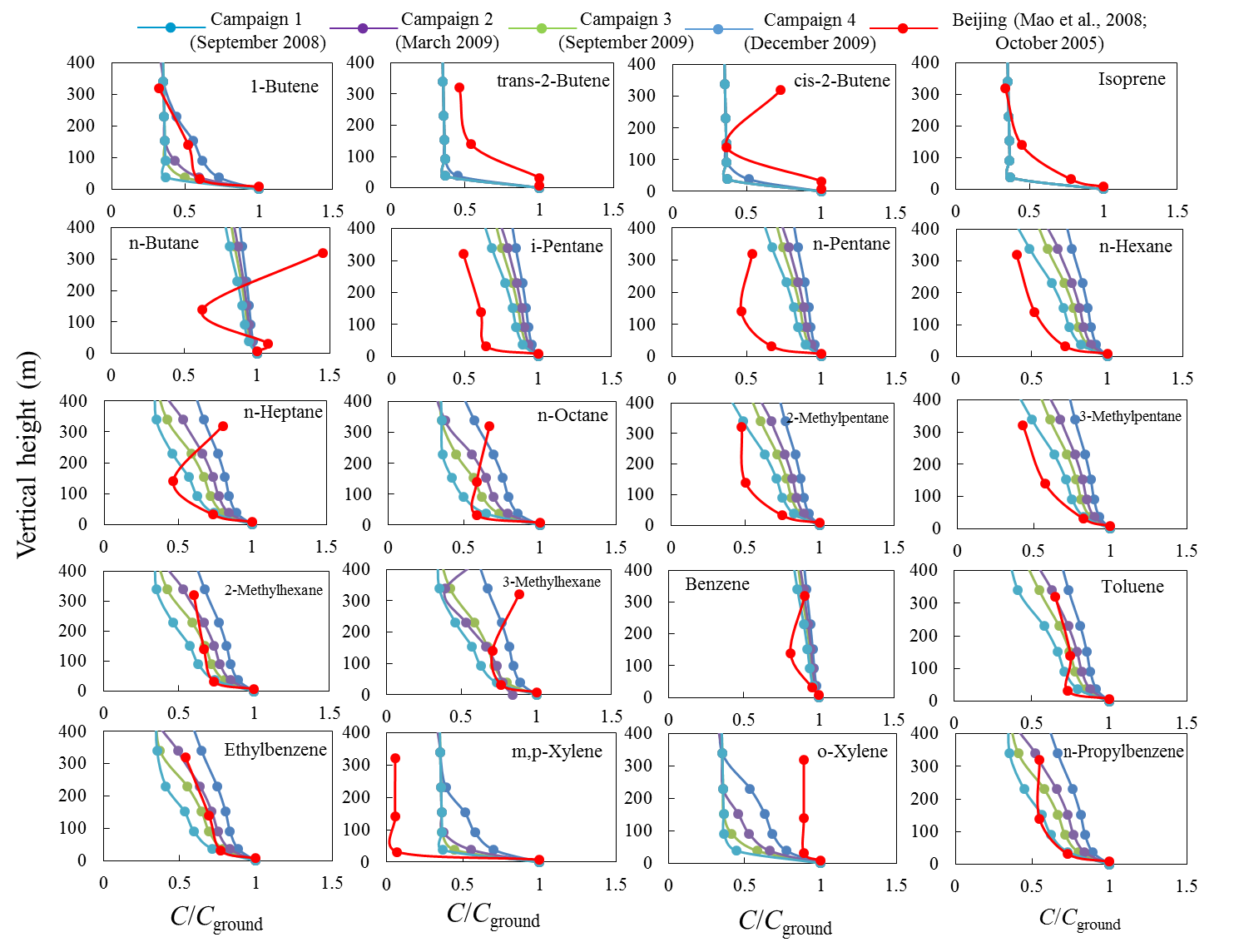


Figure S8. Vertical profiles of VOC species calculated in this work and the results from Mao et al. (2008) in Beijing, China.

­

**Emission ratio method**

The emission ratio method using CO as a reference was used to calculate the VOC emissions, as shown in Equation (S1) (Shao et al., 2011; Zhang et al., 2014):


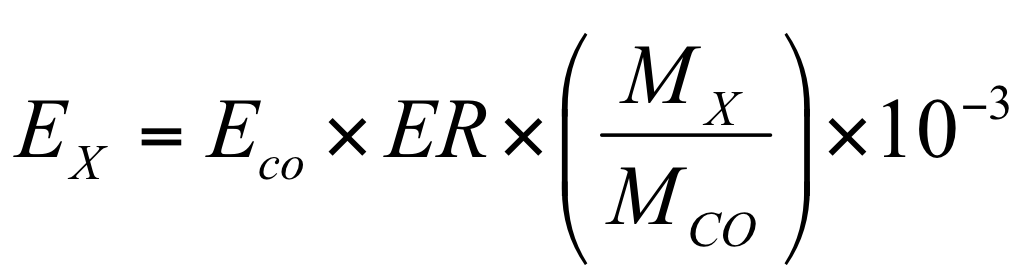
 (S1)

where *E_CO_* is the inventory emissions of CO, *ER* is the slope (ppb/ppm) of the linear correlation between ΔVOC and ΔCO (VOC and CO enhancements above background levels). *M_X_* and *M_CO_* are the molecular weights of each VOC species and CO, respectively. Limitations of this method were mentioned in previous studies (Borbon et al., 2013; Wang et al., 2014) Briefly, (1) the emission ratios for VOCs relative to CO were determined by linear regression fit (using two-sided least-square fit) to calculate the slope between VOC and CO. Here, the ERs reflect the total emissions at the measurement sites, and VOC data at 5:00 am were used to calculate the ERs when photochemical reactions (with OH radical) were at minimum, sources were relatively stable and plumes from individual sources were well-mixed with each other. Noticed that the chemical loss (due to NO_3_ and O_3_) of VOCs at night are assumed to be negligible for the short duration from the source areas to the sites, this might underestimate reactive species like alkenes. Moreover, the uncertainty from linear regression should be considered. (2) Carbon monoxide was selected as the reference tracer in this study, as measured CO showed significant correlations with most anthropogenic NMHC species in urban areas. In addition, CO is an inert species and its emission inventory is well-established in PRD with lower uncertainty (40-50%) compared to other pollutants (Zheng et al., 2009). CO is mainly emitted from combustion-related sources (vehicular exhaust, biomass burning, coal burning), however, some VOCs such as aromatics and alkanes can be emitted from non-combustion sources (solvent use and fuel evaporation sources) in PRD. This assumption would also bias the results. (3) The air masses should represent the average emissions of anthropogenic VOCs in this region, and the composition of VOC emissions does not change much between air masses. (4) The long range transport of air masses does not affect the chemical composition of VOCs at the site. Actually, transport would contribute the observed levels of long-lifetime species (<C3 alkanes and halocarbons), then their ERs at the downwind areas could be overestimated. Therefore, in this work, the VOC/CO method is used as a supplement of VOC estimates for the purpose of comparing the results derived from box models.

The uncertainties of species emissions estimated using CO ratio method were calculated in the following Equation (4):


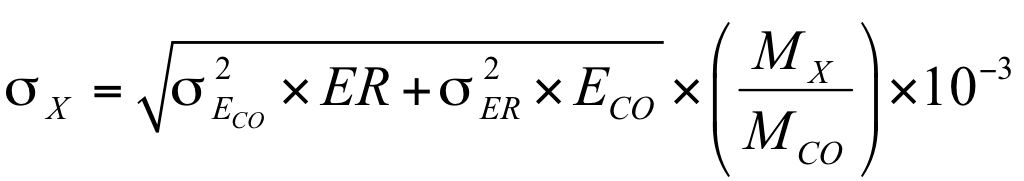
 (S2)

where *σ_Eco_* and *σ_ER_* are the standard deviations of CO inventory emissions and emission ratios, respectively. The emission ratios (*r*^2^ =0.31-0.73) calculated in this work were compared with those in the previous studies, which are listed in Table S8.

**PMF analysis**

The principles and procedures of the model have been described in previous studies (Paatero and Tapper, 1994; Paatero, 1997). An ambient NMHC data set can be viewed as a data matrix *x* of *i* by *j* dimensions, in which *i* number of samples and *j* chemical species are measured. The PMF can identify a number of factors *p*, source profiles *f*, and the amount of mass *g* contributed by each factor to each individual sample, and the residual *e* of each sample/species, as given below in Eq. (S1):

$x_{ij}=\sum_{k=1}^{p} g_{ik}f_{kj}+e_{ij}$ (S1)

The PMF solution minimizes the object function *Q*, based upon the uncertainties:

(S2)

Unusually high NMHC concentrations (probably affected by irregular local emissions) and missing values were replaced by the geometric mean value of all data. Data values below the *MDL* were substituted with *MDL*×1/2. Uncertainty (*u_i_*) was calculated by $u_{i}=\frac{5}{6}MDL$ (MDL is method detection limit; concentration≤ MDL) and $u_{i}=\sqrt{\left(EF\times concentration\right)^{2}+\left(MDL\right)^{2}}$ (EF is the error fraction, specified as precision of the VOC species; concentration > MDL). Missing values and unexpected high concentrations were replaced by the geometric mean values of all data in each event. Data values below the MDL were substituted with MDL×1/2.A total of 30 species were used for these analyses (Table S3). For each of these species, 95% of values recorded were above the detection limit.

The number of factors for the PMF solution was checked from four to ten for the best solution to minimize the object function *Q* (Eq. [S2]). Each test performed 20 base runs to check the stability of the PMF solution, with the lowest *Q* value being selected as the base run solution. The difference between *Q* (true) and *Q* (robust) is a measure of the impact of data points with high scaled residuals (Eq. [S1]). The ratio of *Q* (true) and *Q* (robust) was 1.15, which is acceptable compared to the recommended value of less than 1.5 (Norris et al., 2014). The rotation ambiguity was explored by varying the *F_peak_* values from −5 to 5. The results with *F_peak_* = 0.5 were selected for the lowest *dQ* (robust), indicating the stability of the PMF solution. It should be noted that unlike PMF2, an *F_peak_* value of 0 is not allowed in PMF 5.0 (Norris et al., 2014). The observed mass concentrations of total NMHCs were well resolved by the PMF (*r^2^* = 0.92).

**Reference**

Blake, D. R., Rowland, F. S.. Urban leakage of liquefied petroleum gases and its impact on Mexico City air quality. Science 1995, 269, (5226), 953.

Borbon, A.; Gilman, J.B.; Kuster, W.C.; Grand, N.; Chevaillier, S.; Colomb, A.; Dolgorouky, C.; Gros, V.; Lopez, M.; Sarda-Esteve, R.; Holloway, J.; Stutz, J.; Petetin, H.; McKeen, S.; Beekmann, M.; Warneke, C.; Parrish, D. D.; de Gouw, J.A., Emission ratios of anthropogenic volatile organic compounds in northern mid-latitude megacities: Observations versus emission inventories in Los Angeles and Paris. *J. Geophys. Res.-Atmos.* **2013**, 118 (4), 1-17.

Dumanoglu, Y.; Kara, M.; Altiok, H.; Odabasi, M.; Elbir, T.; Bayram, A., Spatial and seasonal variation and source apportionment of volatile organic compounds (VOCs) in a heavily industrialized region. *Atmos. Environ.* **2014,** *98*, (0), 168-178.

Guenther, A.; Hewitt, C. N.; Erickson, D.; Fall, R.; Geron, C.; Graedel, T.; Harley, P.; Klinger, L.; Lerdau, M.; McKay, W. A.; Pierce, T.; Scholes, B.; Steinbrecher, R.; Tallamraju, R.; Taylor, J.; Zimmerman, P., A global model of natural volatile organic compound emissions. *Journal of Geophysical Research: Atmospheres* **1995,** *100*, (D5), 8873-8892.

Gertler, A. W.; Fujita, E. M.; Pierson, W. R.; Wittorff, D. N., Apportionment of NMHC tailpipe vs non-tailpipe emissions in the Fort McHenry and Tuscarora mountain tunnels. *Atmos. Environ.* **1996,** *30*, (12), 2297-2305.

Liu, Y.; Shao, M.; Fu, L.; Lu, S.; Zeng, L.; Tang, D., Source profiles of volatile organic compounds (VOCs) measured in China: Part I. *Atmos. Environ.* **2008,** *42*, (25), 6247-6260.

Norris, G. A., Duvall, R., Brown, S. G., Bai, S., EPA Positive Matrix Factorization (PMF) 5.0 fundamentals and User Guide Prepared for the US Environmental Protection Agency Office of Research and Development, **2014**, Washington, DC. DC EPA/600/R-14/108.

Ou, J.; Zheng, J.; Li, R.; Huang, X.; Zhong, Z.; Zhong, L.; Lin, H., Speciated OVOC and VOC emission inventories and their implications for reactivity-based ozone control strategy in the Pearl River Delta region, China. *Sci. Total Environ.* **2015**, *530–531*, 393-402.

Paatero, P.; Tapper, U., Positive matrix factorization: a non-negative factor model with optimal utilization of error estimates of data values. *Environmetrics* **1994**, 5, 111-126.

Paatero, P., Least squares formulation of robust nonnegative factor analysis. Chemometrics and Intelligent Laboratory Systems, **1997**, 37, 23-35.

Shao, M.; Huang, D.; Gu, D.; Lu, S.; Chang, C.; Wang, J., Estimate of anthropogenic halocarbon emission based on measured ratio relative to CO in the Pearl River Delta region, China. *Atmos.c Chem. Phys.* **2011**, *11*, (10), 5011-5025.

Watson, J. G.; Chow, J. C.; Fujita, E. M., Review of volatile organic compound source apportionment by chemical mass balance. *Atmos. Environ.* **2001,** *35*, (9), 1567-1584.

Wang, M.; Shao, M.; Chen, W.; Yuan, B.; Lu, S.; Zhang, Q.; Zeng, L.; Wang, Q., A temporally and spatially resolved validation of emission inventories by measurements of ambient volatile organic compounds in Beijing, China. *Atmospheric Chemistry and Physics* **2014,** *14*, (12), 5871-5891.

Yin, S.; Zheng, J.; Lu, Q.; Yuan, Z.; Huang, Z.; Zhong, L.; Lin, H., A refined 2010-based VOC emission inventory and its improvement on modeling regional ozone in the Pearl River Delta Region, China. *Sci. Total Environ.* **2015,** *514*, (0), 426-438.

Yuan, B.; Shao, M.; Lu, S. H.; Wang, B., Source profiles of volatile organic compounds associated with solvent use in Beijing, China. *Atmos. Environ.* **2010,** *44*, (15), 1919-1926.

Yuan, Z.; Zhong, L.; Lau, A. K. H.; Yu, J. Z.; Louie, P. K. K., Volatile organic compounds in the Pearl River Delta: Identification of source regions and recommendations for emission-oriented monitoring strategies. *Atmos. Environ.* **2013,** *76*, 162-172.

Zhang, Y. L.; Wang, X. M.; Simpson, I. J.; Barletta, B.; Blake, D. R.; Meinardi, S.; Louie, P. K. K.; Zhao, X. Y.; Shao, M.; Zhong, L. J.; Wang, B. G.; Wu, D., Ambient CFCs and HCFC-22 observed concurrently at 84 sites in the Pearl River Delta region during the 2008-2009 grid studies. *J. Geophys. Res.-Atmos.* **2014,** *119*, (12), 7699-7717.

Zheng, J. Y.; Shao, M.; Che, W. W.; Zhang, L. J.; Zhong, L. J.; Zhang, Y. H.; Streets, D., Speciated VOC Emission Inventory and Spatial Patterns of Ozone Formation Potential in the Pearl River Delta, China. *Environ. Sci. Technol.* **2009**, *43*, (22), 8580-8586.
